# Guanidino-Aryl Derivatives: Binding to DNA, RNA and G-Quadruplex Structure and Antimetabolic Activity

**DOI:** 10.3390/molecules30183682

**Published:** 2025-09-10

**Authors:** Davor Margetić, Petra Jadrijević-Mladar, Anamaria Brozovic, Lidija-Marija Tumir

**Affiliations:** 1 Division of Organic Chemistry & Biochemistry, Ruđer Bošković Institute, 10000 Zagreb, Croatia; 2 Division of Molecular Biology, Ruđer Bošković Institute, 10000 Zagreb, Croatia; petra.jadrijevic-mladar@irb.hr (P.J.-M.); anamaria.brozovic@irb.hr (A.B.)

**Keywords:** aryl-guanidine, fluorescence, circular dichroism, DNA binding, RNA binding, G-quadruplex

## Abstract

A series of novel guanidino-aryl (**GA**) compounds containing phenanthrene, fluoranthene, fluorene, and naphthalene aromatic cores were synthesized to investigate their interactions with DNA, RNA, and G-quadruplex structures. Among the novel compounds, the phenanthrene-guanidino compound demonstrated the highest micromolar affinity for AT-DNA, possibly due to partial phenanthrene intercalation in addition to hydrogen bonding and electrostatic interactions of guanidine cation. All new guanidino-aryl **GA** compounds bind strongly to the Tel22 G-quadruplex structure with similar affinities regardless of aromatic core size. The 1:1 stoichiometric complex is stabilised by π-π stacking interactions with the top or bottom G-tetrad, together with strong electrostatic interactions of the guanidino cation. The guanidino-porphyrin PoGU displayed distinct binding stoichiometry, indicating possible sandwiching between two G-quadruplex structures. Within the **GA** compounds tested, guanidino-fluorene exhibited moderate antimetabolic activity against the HeLa cell line, without selectivity against the healthy cell line.

## 1. Introduction

DNA and RNA biomacromolecules are involved in critical processes in living cells, including DNA replication, transcription, and processes of protein synthesis [1,2]. Apart from the predominant canonical double helix B-form, polynucleotides adopt various non-canonical structures (NCS), namely triplex, G-quadruplex, i-motif forms, hairpin, cruciform, etc. [3,4].

Numerous biologically active small molecules owe their activity to binding to polynucleotides (namely, intercalation between base pairs, minor or major groove binding, and/or external electrostatic binding [5]). The potential basis of their biological/antimetabolic activity is often their selectivity for certain polynucleotide sequences (AT vs. GC) and/or structures (e.g., single [6] double, or triple-stranded [7] structures, G-quadruplex [8]; a different type of helix, such as A, B, Z, etc.).

Dynamic structural transitions between common B-DNA and non-canonical forms influence and regulate gene expression. Thus, stabilisation of non-canonical structures generated in the parent DNA or RNA by an NCS-selective ligand can enhance or inhibit specific biological activities. As a result, NCS ligands have anticancer and antiviral therapeutic potential [5,9,10,11].

G-quadruplex (**G4**) is a prominent type of non-canonical DNA structure that occurs within guanine-rich regions of the human genome [12]. The **G4** structure consists of four coplanar guanines held together by Hoogsteen hydrogen bonding. Telomeres, non-coding DNA structures at the ends of eukaryotic chromosomes, were discovered to contain repetitive guanine-rich sequences (5′-TTAGGG-3′) [13,14]. G-quadruplex **G4** structures are particularly appealing therapeutic targets since these structures are critical for gene expression, chromosome stability, and recombination [15,16,17,18].

Among the numerous polynucleotide binders that possess biological activity are guanidine-containing compounds. The guanidine functionality is found in important biological molecules: arginine (amino acid), agmatine (neurotransmitter), creatine (muscular energy intermediate) and guanine (purine bases of DNA and RNA) [19]

The protonation of the basic guanidine group leads to the formation of a very stable guanidinium cation, whereby the delocalisation of the guanidinium cation’s positive charge through six π-electrons in the bonding orbitals forms a Y-delocalisation system [20]. Due to its ability for non-covalent interactions, namely, H-bonds, dipole–dipole, and cation–π interactions [21], molecules containing guanine can recognise important biomolecular targets and form complexes with them. Guanidine compounds exert their biological effects mostly through DNA/RNA minor groove binding [22], particularly through various types of non-covalent interactions: hydrogen bonds, van der Waals interactions, and non-specific electrostatic interactions.

Previously, we synthesised and studied a series of guanidino-aryl compounds in which the guanidino group is directly attached to the aryl moiety. Variation in the size of the aryl-moiety (in ascending order: naphthalene, acridine, pyrene and porphyrin) showed weak DNA binding for naphthalene–guanidine with a small condensed aromatic surface, but also no notable difference in DNA affinity for various aromatic surfaces (acridine, pyrene and phenanthridine). Steric caused by the direct attachment of guanidine to the aromatic moiety resulted in binding of the ligands in the minor groove instead of the expected intercalation [23].

**Scheme 1:**
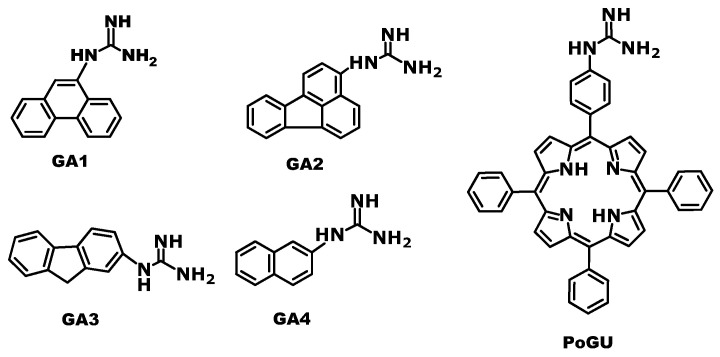
Structures of the studied new aryl-guanidine derivatives **GA1**-**GA4** and **PoGU** [23].

Continuing our previous work, we have prepared a new series of guanidino-aryl molecules, in which the guanidino group is directly bound to an aromatic moiety. For comparison with derivatives with a larger aromatic surface area, a naphthalene derivative was prepared in which the guanidino group is positioned differently from the previously published analogue [23]. We chose aryl moieties with a crescent aromatic shape: phenanthrene, fluorene and fluoranthene. (Scheme 1) The interactions of the new guanidino-aryl (**GA**) compounds with different types of DNA/RNA were investigated using spectroscopic methods, while the previously reported guanidino-porphyrin **PoGU** was also tested together with the new compounds for their affinity to the Tel22 G-quadruplex structure and their antimetabolic activity.

## 2. Results and Discussion

### 2.1. Synthesis

**Scheme 2:**
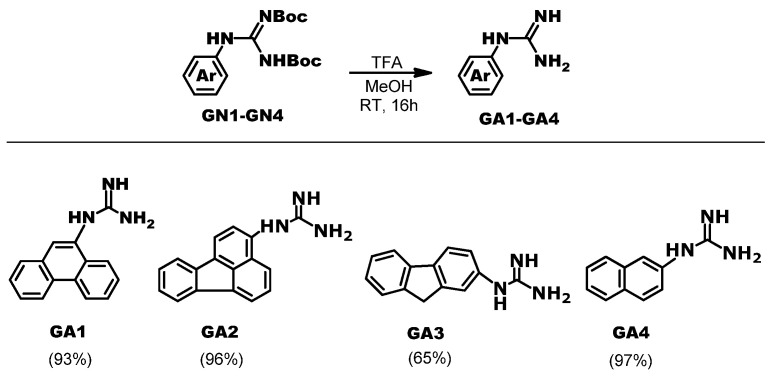
Synthesis of studied aryl-guanidine derivatives **GA1**-**GA4.**

Aromatic guanidines **GA1**-**GA4** were prepared by acidic deprotection of the Boc groups of guanidines **GN1**-**GN4** (Scheme 2). Samples of Boc-protected guanidines **GN1**-**GN4** were available from our previous work [24]. Guanidines **GA3** [25] and **GA4** [26] are known from the literature and have been prepared by different synthetic methods. Porphyrin guanidine **PoGU** was available from our previous study [23].

### 2.2. Spectroscopic Characterisation of ***GA1**-**GA4*** in the Aqueous Medium

Studied compounds **GA1**-**GA4** were dissolved in DMSO (*c* = 1 ×10^−3^ mol dm^−3^) and diluted with buffer for measurements. All measurements were recorded in the Na-cacodylate buffer (*I* = 0.05 mol dm^−3^) at pH = 7.0; the volume ratio of DMSO was less than 1% in all measurements. The p*Ka* value of guanidines is highly dependent on the substituent; however, it varies much less in the aryl-guanidine series. The guanidine group is basic in aryl-guanidine analogues [23,27]. Consequently, the guanidine group was fully protonated at neutral measurement conditions. Absorbancies of aqueous solutions of **GA1**-**GA4** were proportional to their concentrations up to *c* = 2–5 × 10^−5^ mol dm^−3^. Their spectroscopic characterisation is given in the Supplementary Materials (Table S1, Figure S1).

**Figure 1:**
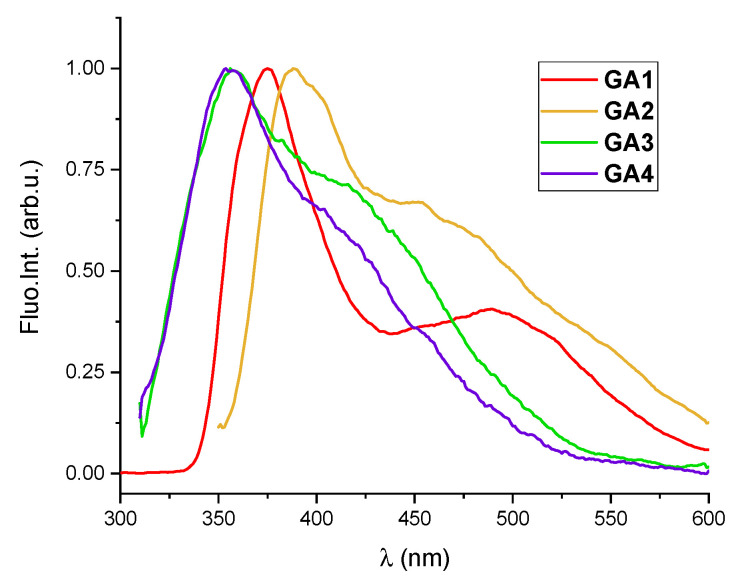
Normalised fluorescence spectra of **GA1** (*c* = 2.5 × 10^−6^ mol dm^−3^; λ_exc_ = 255 nm), **GA2** (*c* = 4.0 × 10^−6^ mol dm^−3^; λ_exc_ = 276 nm), **GA3** (*c* = 1.0 × 10^−6^ mol dm^−3^; λ_exc_ = 267 nm) and **GA4** (*c* = 4.0 × 10^−6^ mol dm^−3^; λ_exc_ = 276 nm) in aqueous solution (Na-cacodylate buffer, *I* = 0.05 mol dm^−3^, pH = 7.0).

Fluorescence emissions of **GA1**-**GA4** (Figure 1) were linearly dependent on their concentrations up to *c* = 4 × 10^−6^ mol dm^−3^. Excitation spectra recorded at emission maxima are in good agreement with UV/Vis spectra.

### 2.3. Interactions of ***GA1**-**GA4*** with DNA and RNA

The affinity of **GA** compounds for double-stranded polynucleotides was investigated. The study used a variety of natural (calf-thymus DNA with 58% AT and 42% GC base pairs) and synthetic polynucleotides to investigate the eventual selectivity of **GA** compounds for several well-defined structural features with varying base compositions, groove size, and shape. Natural *ct*-DNA, synthetic alternating poly dAdT-poly dAdT and poly dGdC-poly dGdC represented typical B-helical structures, while the A-helical structure was represented by RNA poly rA–poly rU [2,28]. AT sequences are more flexible than GC-sequences, with narrower minor grooves. This deep and narrow minor groove in the AT region with a series of hydrogen bond acceptors (adenine N3 and thymine O2) is suitable for typical crescent-shaped minor groove binders (like netropsin, Hoechst 33258) [29,30,31]. GC sequences have a shallower groove compared to AT sequences due to the exocyclic guanine N2 substituent. Further, GC pairs are more electron-rich. Therefore, electron-deficient planar aromatic molecules tend to intercalate preferentially between GC base pairs [30]. Structural features of the polynucleotides used in the study are given in Supplementary Materials (Table S2).

Disclosing the binding mode of small molecules to DNA is crucial for comprehending the underlying physical–chemical processes and their prospective biological applications. For example, alkaloid palmatine, a DNA binder and candidate for photodynamic therapy, contains a tetracyclic ring system and a positive charge, and has the capacity for both intercalation and groove binding. Long-range molecular dynamics simulations identified that intercalation is the dominant binding mode of palmatine with DNA [32]. Further, aromatic molecules such as anthracene, pyrene, and porphyrin typically bind to polynucleotides via intercalation. However, direct attachment of the guanidine moiety to the aryl core in previously studied guanidino-aryl series caused steric hindrance and, as a result, minor groove binding rather than intercalation [23].

#### 2.3.1. Thermal Melting Studies

Small-molecule binding to double-stranded DNA/RNA generally influences double-helix stability. The stabilisation or destabilisation of a polynucleotide was indicated by a change in its melting temperature, at which the double helix dissociates into two single-stranded chains (*Tm*) [33]. Thermal melting experiments showed that **GA1**-**GA4** had a small to negligible effect on double helix stability both for DNA and RNA (Table 1, Supplementary Materials, Figure S2). Only **GA1** (phenanthrene compound) and **GA4** (naphthalene compound) showed a small stabilisation effect on *ct*-DNA double helix, while **GA1** also slightly stabilised poly dAdT-poly dAdT and RNA poly rA-poly rU.

The lack of double-helix stabilisation for **GA2** (fluoranthene) and **GA3** (fluorene) compounds rules out classical intercalation as a major binding method [34]. These findings are consistent with previous research, since intercalative binding was found for phenanthrene [35,36] and naphthalene [37,38] compounds, but not for fluoranthene [39] and fluorene [38], due to weak stacking interactions and lack of polarizability. Furthermore, the limited stabilisation impact of **GA1** and **GA4** also does not support complete intercalation of aromatic moieties because guanidine groups directly attached to aryl induce steric hindrance that limits adjustment between base pairs.

#### 2.3.2. Fluorimetric Titrations

All aryl-guanidino compounds **GA1**-**GA4** exhibited a change (increase or quenching) of their intrinsic fluorescence spectra upon the addition of various DNA/RNA. At ratios with an excess of polynucleotide over compound (*r*_[compound]/[polynucleotide]_ < 0.3), spectral shifts might be related to one dominant binding mode. The titration data were analysed using the Scatchard equation [40,41] to determine the stability constants and ratios *n*_[bound compound]/[polynucleotide]_ (Table 2, Supplementary Materials, Figures S4–S19).

**Figure 2:**
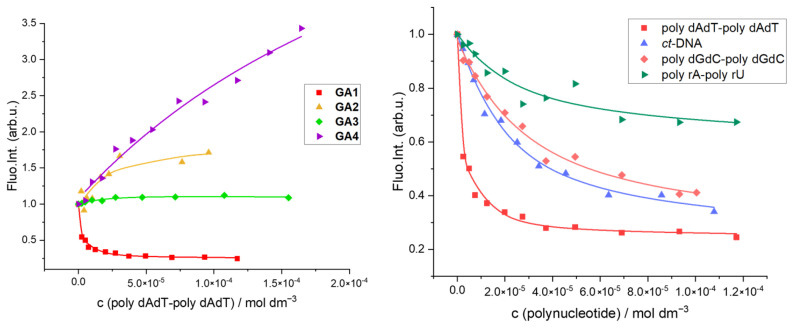
(**Left**) Normalised changes in fluorescence emission of **GA1**-**GA4** (*c* = 2.5 × 10^−6^ mol dm^−3^) upon addition of poly dAdT-poly dAdT at pH 7 (Na-cacodylate buffer, pH = 7.0, *I* = 0.05 M; *λ_exc_* = 300–325 nm); (**Right**) Normalised changes in fluorescence emission of **GA1** (*c* = 2.5 × 10^−6^ mol dm^−3^) upon addition of *ds*-polynucleotides at pH 7 (Na-cacodylate buffer, pH = 7.0, *I* = 0.05 M; *λ_exc_* = 300 nm); polynucleotide concentration was expressed as concentration of phosphates (nucleotides).

Fluorescence of phenanthrene-9-guanidino compound **GA1** was quenched by 30–70% upon addition of all tested polynucleotides. In contrast, the fluorescence of compounds **GA2**-**GA4** increased with titration, but the changes were mostly too small to accurately determine the stability constant. (Table 2, Figure 2, Left). Differences in the type of emission responses of the examined compounds **GA1**-**GA4** are caused by the intrinsic properties of each chromophore and cannot be attributed to the binding affinity of the binding site type, including polynucleotide secondary structure or sequence (Supplementary Materials, Table S2).

Phenanthrene-guanidino **GA1** showed the strongest affinity for polynucleotides compared to other aryl-guanidino compounds, whereby the highest affinity was found for alternating poly dAdT-poly dAdT (Table 2, Figure 2, Right). Despite a significant fluorescence increase, naphthalene derivative **GA4** has the lowest calculated affinity for B-DNA polynucleotide poly dAdT-poly dAdT among other **GA** compounds. The lower affinity and thermal melting results of **GA4** are consistent with a partial intercalative binding mode. This is a consequence of the smaller aromatic surface and weaker π-π aromatic interaction of naphthalene compared to phenanthrene **GA1** (Table 2).

#### 2.3.3. Circular Dichroism Experiments

Circular dichroism (CD) spectroscopy was used to detect conformational changes in polynucleotide secondary structure caused by small-molecule binding. The characteristic circular dichroism (CD) spectrum of DNA B-form has a positive band at 275 nm attributed to base stacking and a negative band at 245 nm caused by helicity. While groove binding and electrostatic interactions of small molecules do not change significantly base stacking and helicity bands, intercalation has a larger impact on the intensities of both bands [35,42]. Furthermore, when an achiral ligand interacts with DNA, it can acquire an induced CD (ICD) signal by coupling its electronic transition moments with the DNA bases. An ICD signal observed within the achiral ligand’s absorption bands indicates a ligand–DNA interaction, characterised by the consistent orientation of the ligand relative to the helix and pseudo dyad axis. The intensity and sign of the ICD signal point to the mode of interaction (intercalation, groove binding, agglomeration, etc.) [43,44,45,46].

In most circular dichroism (CD) titrations of polynucleotides with **GA** compounds, changes in polynucleotide spectra were small or negligible. CD titration results do not clearly identify the specific binding modes of ligands to polynucleotides. Observed changes in CD spectra are ambiguous because **GA1**-**GA4** mostly absorbed UV light below 290 nm, which is the same wavelength region as for polynucleotides. If a ligand absorbs UV light within the same range as polynucleotide, its eventual ICD (positive or negative, depending on the binding mode and orientation of the ligand’s chromophore relative to the helix axis), will be correlated with polynucleotide’s intrinsic CD spectra [47]. Thus, every change in CD spectra can be caused by a polynucleotide helicity change or by induced CD spectra of the ligand or a combination of these events. Further, changes in opposing signs and shifts (for example, a decrease in polynucleotide signal and positive induced CD spectra of the compound) can cancel each other out.

No significant changes in the CD spectra were observed during the titration of poly rA-poly rU with **GA1**-**GA4** (Supplementary Materials, Figures S25–S30). CD spectra of poly dAdT-poly dAdT showed a decrease in both positive and negative bands upon addition of **GA1**, with a small bathochromic shift in CD maxima at 260 nm (Supplementary Materials, Figure S25 Left). It is not possible to distinguish each separate contribution, i.e., a distortion of polynucleotide helicity upon intercalation of phenanthrene and eventual induced negative CD spectra caused by the uniform orientation of **GA1** dipoles regarding the helix axis. The CD spectra of poly dAdT-poly dAdT showed a small increase in the bands upon addition of **GA2**-**GA4** compounds.

**Figure 3:**
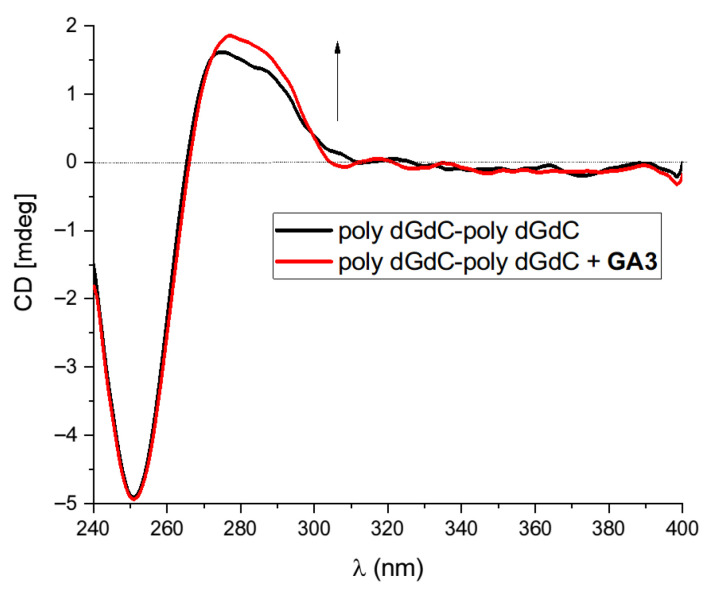
Changes in the CD spectrum of poly dGdC-poly dGdC (*c*(DNA) = 3 × 10^−5^ mol dm^−3^) upon addition of **GA3** at molar ratios *r* = 0.5; *r* = [compound]/[polynucleotide], pH = 7.0, sodium cacodylate buffer and *I* = 0.05 mol dm^−3^.

A minor groove of poly dGdC-poly dGdC is not suitable for groove binding because of the hinderance of protruding NH_2_ groups [30]; therefore, partial intercalation or electrostatic binding is possible for GC polynucleotides. Poly dGdC-poly dGdC CD spectra did not change after the addition of **GA2** and **GA4**, while they showed a slight increase in the case of **GA1** and **GA3** (Figure 3).

#### 2.3.4. Discussion of the Spectroscopic Results

Our objective was to investigate the impact of various aryl groups attached to positively charged guanidine on its binding affinity to DNA/RNA. The guanidino group directly linked to the aryl prevents the complete intercalation of planar aryl groups between base pairs [23]. On the other hand, the planar positively charged guanidino group contributes to noncovalent interactions.

Guanidino cations contain 6π electrons in the bonding orbitals, and their delocalisation is called “Y aromaticity” [21,22]. These electrons are available for hydrogen bonding interactions, cation–π interactions, and strong electrostatic interactions with the negatively charged phosphate backbone.

Among **GA** compounds, the phenanthrene compound **GA1** exhibited the highest affinity for DNA/RNA among the investigated compounds. **GA1** showed small thermal stabilisation, alternating AT polynucleotide and micromolar affinity obtained from fluorimetric titration. Classical intercalation can be excluded according to the small stabilisation effect [34]. But there is a possibility of partial intercalation of the planar aromatic phenanthrene moiety, which is consistent with fluorescence quenching [35]. Guanidine cations could contribute to binding via hydrogen bonding and electrostatic interactions. The comparison of **GA4** and **GA1** binding constants suggests aromatic binding interactions in **GA4**, as the affinity is two orders of magnitude lower, which is consistent with naphthalene’s smaller aromatic surface. Yet, without an unambiguous ICD signal from CD titration data, any definitive conclusion about the binding mode remains hypothetical.

It is reported that the possibility of intercalation of fluorene is dependent upon regiochemical factors [38]. Intercalation was found for benzofluoranthene [48], but not for fluoranthene on gold nanoparticles [39]. However, in the case of **GA2** and **GA3**, the binding mode remains unclear due to small stabilisation effects and small spectroscopic changes.

Although CD titration spectroscopy is generally beneficial, it cannot be used to determine the binding mode for **GA** compounds. Effects in polynucleotide CD spectra were minor or negligible in all titrations, and the changes were ambiguous due to polynucleotide and **GA** ligand absorption spectra overlap.

### 2.4. Interactions of ***GA1**-**GA4*** and ***PoGU*** with G-Quadruplex

G-quadruplex (**G4**) is a prominent kind of non-canonical DNA structure that occurs within guanine-rich regions of the human genome [12]. **G4** structures form within DNA as well as within RNA sequences. This structure consists of four coplanar guanines held together by Hoogsteen hydrogen bonding [49]. The non-canonical structures of **G4** are being extensively examined as therapeutic targets for anticancer and antiviral medications, while **G4** ligands have potential in precision medicine or as additions to existing drugs.

Telomeric DNA in vertebrates has a repeated sequence d(TTAGGG); therefore, oligonucleotide Tel22 (5′-AGGG(TTAGGG)3–3′) is commonly used as a model of the G-quadruplex structure. We investigated interactions between novel compounds **GA1**-**GA4** with Tel22 by fluorimetric and CD titrations and thermal melting experiments in Na-cacodylate buffer (pH = 7, *I* = 0.1 M). Under these conditions (presence of sodium ions), Tel22 is intramolecularly folded into a basket-type structure: antiparallel–parallel stranded G-quadruplex structure with three stacked G-tetrads joined by two lateral loops and a central diagonal loop [50]. Porphyrin compound TMPyP4 is a well-known telomerase inhibitor and quadruplex ligand that can induce the formation of a quadruplex structure from single-stranded DNA [51,52,53]. Therefore, we included the previously published guanidino-porphyrin derivative **PoGU** [23] in the study of interactions with G-quadruplex.

Studied compounds did not show stronger stabilisation of Tel22 G-quadruplex structure compared to double-stranded polynucleotides (Table 1; Supplementary Materials, Table S3, Figure S3). Moreover, only the naphthalene compound **GA4** showed weak stabilisation of the G-quadruplex structure.

**Figure 4:**
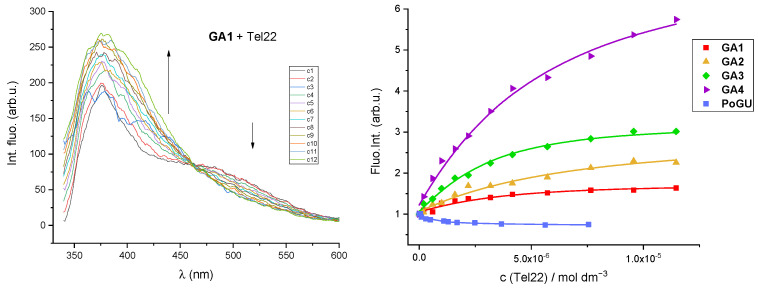
(**Left**) Fluorimetric titration of **GA1**, λ_exc_ = 300 nm and *c* = 2.5 × 10^−6^ mol dm^−3^ with Tel22. (**Right**) Normalised changes in fluorescence emission of **GA1**-**GA4** and **PoGU** (*c* = 1–2.5 × 10^−6^ mol dm^−3^) upon addition of Tel22 G-quadruplex structure at pH 7 (Na-cacodylate buffer, pH = 7.0, *I* = 0.10 M; *λ_exc_* = 300–325 nm for **GA** and *λ_exc_* = 420 nm for **PoGU**).

Results of fluorescence titrations revealed strong affinities of all examined compounds toward the Tel22 G-quadruplex structure. Addition of Tel22 caused a strong fluorescence increase of **GA1**-**GA4** compounds, while emission of **PoGU** decreased upon titration with Tel22 (Figure 4, Right). Stability constants and stoichiometries of guanidinoaril-Tel22 complexes were calculated using the non-linear least-square program SPECFIT Version 2.11 [54,55,56] (Table 3, Supplementary Materials, Figures S20–S24). For **GA1**-**GA4** compounds, the best fit was found for 1:1 **GA1**-**GA4**–Tel22 stoichiometry, and calculation gave very similar constants for all **GA1**-**GA4**–Tel22 complexes. Coexistence of two spectroscopically active species (free ligand and **GA1**–Tel22 complex) and 1:1 stoichiometry is further emphasised by the appearance of the isosbestic point at *λ* = 463 nm (Figure 4, Left). Titration data obtained for the **PoGU**–Tel22 complex did not fit well for 1:1 stoichiometry. The calculation suggested the coexistence of 1:1 and 1:2 stoichiometries; the latter was obtained at a higher excess of Tel22. According to this result, **PoGU** was likely incorporated between two G-quadruplex structures and the complex was stabilised by strong π-π interactions between the large porphyrin core and G-tetrades [57,58].

Examined compounds mostly showed negligible or no influence on the CD spectra of Tel22 (Supplementary Materials, Figures S31 and S32). In the case of **GA1**-**GA4,** eventual ICD signals could possibly appear in the UV region below 290, which overlaps with the polynucleotide signal itself (see Section 2.3.3.). **PoGU** had a strong absorbance at 400 nm, but no ICD signal was observed in this wavelength region. In the case of **GA1,** a small increase in the quadruplex CD signal occurred, while titration with **PoGU** resulted in a small decrease in the quadruplex CD band. **GA1**-**GA4** caused a lack of thermal stabilisation of the quadruplex structure, negligible CD changes and showed high binding affinities for Tel22. **GA1**-**GA4** compounds with different aromatic surfaces showed similar affinities (see Table 3), independent of ligand aromatic core size. Most probably, **GA1**-**GA4** bind to Tel22 on the top or bottom Tel22 G-tetrad through a combination of π-π interactions of aryl contributed by guanidino group interactions. The similar affinity of all **GA** compounds for Tel22, regardless of the aromatic surface of the aryl component, strongly suggests that the interaction of the **GA** ligand’s guanidino cation with the oligonucleotide, either through hydrogen bonding or nonspecific electrostatic interactions, is dominant in stabilising the complex. Such binding does not affect Tel22 structure or helicity. In the case of **PoGU**, CD results and thermal melting are consistent with high stability constants (Table 3) and possible binding of a large aromatic guanidino-porphyrin in the sandwich between two G-quadruplex structures.

### 2.5. Biological Activity of ***GA1***-***GA4*** and ***PoGU***

The guanidine group occurs in a variety of biologically important compounds, making guanidine compounds important in medicinal chemistry. Guanidine-containing derivatives have a broad spectrum of biological activities, including anti-inflammatory, Na+/H+ exchanger inhibitory, NO synthase inhibitory, antithrombotic, antidiabetic, antiviral, antiparasitic, and chemotherapeutic properties [22,59].Some of the guanidine compounds are currently used as anticancer drugs [19,59]. Many of the guanidine compounds owe their antitumor activity to non-covalent DNA interactions, especially binding to the minor grooves [22]. Here, we tested the guanidino-aryl compounds **GA1**-**GA4** and **PoGU** for their effect on HeLa (human cervical adenocarcinoma) cells and human skin fibroblasts’ metabolic activity as an indirect measure of cell viability. Compounds **GA1**-**GA3** and **PoGU** [23] showed similar affinity for DNA/RNA, while all **GA1**-**GA4** and **PoGU** showed high affinity for the Tel22 G-quadruplex structure. Despite similar affinities of compounds for DNA/RNA, the results revealed a negligible effect of all investigated derivatives on the metabolic activity of the two different cell lines, except for the fluorene derivative GA3 (Supplementary Materials, Figures S34 and S35), which showed a biological effect (*IC*_50_ 76 µM ± 2.3 for HeLa cells and 26 µM ± 3.1 for fibroblasts) comparable to some cyclometalated Ir(III) complexes containing guanidinium groups as ligands [60].

Besides DNA binding as the most important mechanism, there are also other various cytotoxicity mechanisms described, such as interference with cell membranes, reactive oxygen species (ROS) generation, mitochondrial-mediated apoptosis, Rac1-mediated inhibition of the MAPK/ERK signalling pathway, and induction of cellular apoptosis [19,22,61].

The DNA-binding affinity of guanidine-containing compounds is usually positively correlated with their antiproliferative activity [19]. However, there are also examples that the weakest DNA binders among the investigated derivatives exhibit the most effective cytotoxic and apoptosis-inducing properties. This suggests that the guanidino derivatives may induce apoptosis by a mechanism other than DNA binding. Such guanidino-aryl compounds have been found to inhibit protein kinases [22]. Based on our data, which show a weaker DNA binding capacity of **GA3** compared to **GA1**, and the literature data, the antimetabolic toxic effect of **GA3** does not appear to be due to DNA binding.

## 3. Materials and Methods

### 3.1. Synthesis of Guanidino-Aryl Compounds ***GA1**-**GA4***

#### 3.1.1. General Procedure: Synthesis and Characterisation

Commercially available chemicals and solvents were used without prior purification. Fine organic chemicals were purchased from BLDpharm, TCI and Alfa Æsar, and solvents from KEFO (EtOH, MeOH, and EtOAc), Gram-Mol (petroleum ether, b.p. 65–90 °C and CH_2_Cl_2_), or Riedel-de-Haën (CH_3_CN). TLC plates (TLC Silicagel 60, 63–200 µm, 254 nm) were purchased from Merck KGaA. NMR spectra were recorded on Bruker Avance AV600 and AV300 (Bruker Corporation, Billerica, MA, USA) using tetramethylsilane (TMS) as the reference, and the coupling constants (*J*) are expressed in Hertz (Hz). Multiplicities in ^1^H-NMR spectra are denoted as follows: s—singlet; d—doublet; dd—doublet of doubles; t—triplet; m—multiplet; bs—broad signal. High-resolution mass spectra (HRMS) were recorded on an Agilent 6550 Series Accurate-Mass-Quadrupole Time-of-Flight (Q-TOF) instrument with Electrospray Ionisation (ESI) (Agilent Technologies, Santa Clara, CA, USA)

#### 3.1.2. General Procedure for Boc Deprotection

To a solution of Boc-substituted guanidine (20 mg) in methanol (0.5 mL), trifluoroacetic acid (0.5 mL) was added, and the mixture was stirred at room temperature for 16 h. The solvent was removed in vacuo, and the residue was basified with aqueous Na_2_CO_3_, and then extracted with dichloromethane and ethyl acetate. Organic extracts were combined, and the solvent was removed in vacuo to obtain deprotected guanidine.

**GA1 1-(phenanthren-9-yl)guanidine:** pale yellow amorphous solid, m.p. > 280 °C Yield 93%; ^1^H NMR (CDCl_3_) δ/ppm: 8.67 (d, 1H, *J* = 8.0 Hz), 8.62–8.59 (m, 1H), 8.18 (dd, 1H, *J* = 8.1, 1.1 Hz), 7.75–7.70 (m, 1H), 7.63 (dt, 1H, *J* = 7.3, 1.1 Hz), 7.59–7.50 (m, 3H), 7.24 (brs, 3H), 4.51 (brs, 1H, NH); ^1^H NMR (DMSO-*d*_6_) δ/ppm: 8.84 (d, 1H, *J* = 8.0 Hz), 8.79–8.74 (m, 1H), 8.14 (dd, 1H, *J* = 8.1, 1.1 Hz), 7.91–7.85 (m, 1H), 7.74–7.64 (m, 2H), 7.61–7.56 (m, 2H), 7.45 (brs, 1H), 6.33 (brs, 3H, NH); ^13^C NMR (CDCl_3_) δ/ppm: 153.27, 141.36, 132.91, 131.70, 129.63, 128.50, 127.92, 127.22, 127.07, 126.89, 125.59, 124.56, 123.09, 122.81, 118.68; HRMS ESI-Q-TOF: Found: [M]^+^: 236.1190 calculated for [C_15_H_15_N_3_]^+^, [M + H]^+^: 236.1188. (Supplementary Materials, Figures S36–S39).

**GA2 1-(fluoranthen-3-yl)guanidine:** pale yellow amorphous solid, m.p. > 280 °C Yield 96%; ^1^H NMR (DMSO-*d*_6_) δ/ppm: 8.08 (d, 1H, *J* = 6.9 Hz), 8.01 (d, 1H, *J* = 2.5 Hz), 7.99 (brs, 1H), 7.93 (d, 1H, *J* = 7.7 Hz), 7.91 (d, 1H, *J* = 8.9 Hz), 7.58 (dd, 1H, *J* = 8.6, 7.7 Hz), 7.37 (dd, 1H, *J* = 8.2, 7.2 Hz), 7.30 (dd, 1H, *J* = 7.9, 7.5 Hz), 6.99 (d, 1H, *J* = 7.4 Hz), 5.52 (brs, 4H, NH); ^13^C NMR (DMSO-*d*_6_) δ/ppm: 154.23, 150.84, 140.11, 138.98, 136.57, 133.86, 128.32, 128.22, 127.67, 126.81, 126.49, 125.01, 123.44, 122.46, 121.31, 121.11, 119.63; HRMS ESI-Q-TOF: Found: [M]^+^: 260.1190, calculated for [C_17_H_13_N_3_]^+^, [M + H]^+^: 260.1188. (Supplementary Materials, Figures S40–S42).

**GA3 1-(9H-fluoren-2-yl)guanidine:** pale yellow amorphous solid, m.p. 205–207 °C Yield 65%; ^1^H NMR (DMSO-*d*_6_) δ/ppm: 7.74 (d, 1H, *J* = 7.6 Hz), 7.70 (d, 1H, *J* = 7.9 Hz), 7.51 (d, 1H, *J* = 7.1 Hz), 7.33 (dd, 1H, *J* = 8.7, 7.0 Hz), 7.20 (d, 1H, *J* = 9.0, 6.5 Hz), 7.04 (brs, 1H), 6.83 (d, 1H, *J* = 8.2 Hz), 5.47 (brs, 3H, NH), 3.82 (s, 1H, CH_2_); ^13^C NMR (DMSO-*d*_6_) δ/ppm: 153.44, 144.96, 143.27, 142.66, 134.81, 127.51, 126.13, 125.97, 125.79, 122.72, 121.17, 120.47, 119.71, 37.23 (CH_2_); HRMS ESI-Q-TOF: Found: [M]^+^: 224.1190, calculated for [C_14_H_13_N_3_]^+^, [M + H]^+^: 224.1188. (Supplementary Materials, Figures S43–S45).

**GA4 1-(naphthalen-2-yl)guanidine:** pale yellow amorphous solid, m.p. 146–148-150 °C Yield 97%; ^1^H NMR (DMSO-*d*_6_) δ/ppm: 7.78–7.68 (m, 3H), 7.38 (dd, 1H, *J* = 8.1, 7.2 Hz), 7.29 (d, 1H, *J* = 7.6 Hz), 7.24 (brs, 1H), 7.08 (dd, 1H, *J* = 8.7, 1.7 Hz), 5.61 (brs, 4H, NH); ^13^C NMR (DMSO-*d*_6_) δ/ppm: 153.86, 148.32, 135.39, 129.76, 129.13, 128.21, 127.38, 126.48, 126.06, 123.96, 118.67; HRMS ESI-Q-TOF: Found: [M]^+^: 186.1030, calculated for [C_11_H_11_N_3_]^+^, [M + H]^+^: 186.1031. (Supplementary Materials, Figure S46–S48).

### 3.2. General Procedures for the Study of DNA/RNA Interactions

UV–Vis absorption spectra of compounds were measured on a Varian Cary 100 Bio spectrometer (Agilent, Santa Clara, CA, USA). Thermal melting experiments were measured on a JASCO V-730 UV–Vis spectrometer (ABL&E Handels GmbH, Wien, Austria). Fluorescence spectra and fluorimetric titrations were measured on a Varian Cary Eclipse fluorimeter (Agilent, Santa Clara, CA, USA). CD spectra were measured on a JASCO J815 spectrophotometer (ABL&E Handels GmbH, Wien, Austria). UV–Vis, fluorescence and CD spectra were recorded in the suitable 1 cm path quartz cuvettes.

Polynucleotides were bought, as stated: calf thymus (*ct*)-DNA, poly dAdT-poly dAdT, poly dGdC-poly dGdC and poly rA-poly rU (Sigma-Aldrich Darmstadt, Germany). They were dissolved in sodium cacodylate buffer, where *I* = 0.05 mol dm^−3^ and pH = 7.0. The calf thymus *ct*-DNA was also sonicated and filtered with a 0.45 µm filter [62]. The polynucleotide concentration was evaluated spectroscopically and presented as the concentration of phosphates [63,64]. Oligonucleotide 5′-AGGG(TTAGGG)3–3′ (Tel22) was purchased from IDT Integrated DNA Technologies USA (Leuven, Belgium). Tel22 was dissolved in 0.1 M sodium cacodylate buffer. The initial oligonucleotide solution was first heated up to 95 °C for 10 369 min followed by slowly cooling to 10 °C at a cooling rate of 1 °C/min to enable the oligonucleotide to form a G-quadruplex structure [49,65]. The antiparallel basket-type G-quadruplex structure characteristic for the presence of sodium ions was verified by thermal melting and CD spectra [65,66,67]. The concentration of G-quadruplex is defined in terms of oligonucleotide structure. Stock solutions of guanidine-aryl compounds were prepared by dissolving the compounds in DMSO; total DMSO content was less than 1% in UV–Vis and less than 0.1% in fluorimetric measurements. All measurements were performed in sodium cacodylate buffer, where *I* = 0.05 M or 0.1 M, pH = 7.0. **GA1**-**GA4** concentrations below 1 × 10^−5^ M were used for UV–Vis absorbance measurement to avoid intermolecular association.

### 3.3. CD and Fluorescence Titrations

Fluorimetric titrations were conducted by introducing aliquots of polynucleotide or oligonucleotide solution into the solution of the compound studied. Every addition of an oligonucleotide or polynucleotide aliquot resulted in equilibrium in less than 120 s. In fluorimetric titrations, the concentration of **GA1**-**GA4** and **PoGU** compounds was 1–2 × 10^−6^ M; emission and excitation slits were 20 nm for **GA** compounds and 10 nm for **PoGU**. The excitation wavelength of *λ_exc_* > 300 nm was selected to avoid absorption of excitation light induced by increased absorbance of the polynucleotide. Emission was reported in the range *λ_em_* = 330–600 nm. We collected fluorescence spectra at *r* < 0.3 (*r* = [compound]/[polynucleotide]) to ensure one dominant binding mode. Titration data for *ds*-DNA and *ds*-RNA were processed using the Scatchard equation [40,41]. Calculations generally provided values of ratio *n* = 0.2 ± 0.05; however, to facilitate comparison, all Ks values were recalculated for a fixed *n* = 0.2. *Ks* values show satisfactory correlation coefficients (>0.98). The binding constants and stoichiometries of **GA1**-**GA4** and **PoGU**–Tel22 complexes were computed via multivariate, non-linear, least square regression analysis using the SPECFIT program Version 2.11 [54,55,56]. The concentration range is related to 20–80% complex formation. CD studies were carried out by introducing aliquots of stock solutions of compound **GA1**-**GA4** and **PoGU** into solution of the polynucleotides or oligonucleotide (*c* ≈ 1–2 × 10^−5^ M). Compounds **GA1**-**GA4** and **PoGU** are achiral and so do not have intrinsic CD spectra. CD spectra were obtained at a scan rate of 200 nm/min. Each spectrum was subjected to two accumulations, and the buffer background was subtracted.

### 3.4. Thermal Denaturation Experiments

The thermal denaturation curves for *ds*-DNA, *ds*-RNA, Tel22, and their complexes with chemicals **GA1**-**GA4** and **PoGU** were obtained by tracking the absorbance change at 260 nm as a function of temperature [33]. The heating rate was 1 °C/min. The polynucleotide concentration was 2 × 10^−5^ M (expressed per nucleotide), with *r* = 0.3 (*r* = [compound]/[polynucleotide]). The Tel22 concentration was 1–2 × 10^−6^ M (expressed as oligonucleotide concentration), with the *r*_[compound]/[Tel22]_ = 4. The absorbance scale was normalised. *Tm* values are the midpoints of the transition curves calculated from the maximum of the first derivative and graphically verified using the tangent method. The Δ*Tm* values were derived by subtracting the free nucleic acid’s *Tm* from that of the complex. Every Δ*Tm* value presented here is the average of at least two measurements. The inaccuracy in Δ*Tm* is ±0.5 °C.

### 3.5. Evaluation of the Metabolic Activity

The cell metabolic activity was assessed by MTT assay [68] modified accordingly. Examined **GA1**-**GA4** and **PoGU** compounds were dissolved in DMSO (1 × 10^−2^ mol dm^−3^) and kept as stock solutions at 4 °C.

#### Cell Lines

The experiments were performed with HeLa cells (human cervical adenocarcinoma; purchased from the cell culture bank (GIBCO BRL-Invitrogen, Grand Island, NY, USA)) and human skin fibroblast, as described previously [69].

Cells were cultured in Dulbecco’s modified Eagle medium—DMEM (Gibco, Paisley, Scotland) supplemented with 10% heat-inactivated fetal bovine serum (FBS, Gibco, Paisley, Scotland), 2 mM glutamine, and 100 U/0.1 mg penicillin/streptomycin. Cells were grown at 37 °C in a humidified atmosphere with 5% CO_2_.

Cells were plated in 96-well flat-bottom plates (at a concentration of 5 × 10^3^ cells/180 μL per well) and 24 h later, they were treated with different concentrations of compounds and incubated for 72 h. The working dilutions were freshly prepared in the media on the day of testing. The solvent was also tested for any inhibitory effect by adjusting its concentration to match that of the working concentrations. After 72 h of incubation, the metabolic activity of the cells was evaluated using the MTT assay [68].

The calculation of the effect of the compounds tested on cell metabolic activity has been described previously [69].

## 4. Conclusions

Novel guanidino-aryl compounds (**GA**) with varying aromatic scaffolds were synthesized and evaluated for their binding affinities toward double-stranded DNA/RNA polynucleotides and G-quadruplex structures. All tested compounds (**GA1**-**GA4**) bound to double-stranded polynucleotides and exhibited different fluorescence changes upon titration with nucleic acids, where a different fluorimetric response was influenced by the nature of the aromatic chromophore and not correlated with the polynucleotide sequence or secondary structure of the polynucleotide.

The phenanthrene-based compound **GA1** demonstrated the highest affinity for DNA/RNA among investigated compounds, particularly for the alternating AT-DNA sequence. The potential binding mode, supported by fluorescence quenching, involves partial phenanthrene intercalation in addition to hydrogen bonding and electrostatic interactions of guanidine cation. Definitive conclusion about the binding mode remains hypothetical without the support of CD spectroscopy results.

CD titrations predominantly demonstrated small or negligible alterations of polynucleotide spectra and an absence of ICD spectra, while the absorption spectra of polynucleotides and GA ligands overlapped, so the CD titration outcomes were insufficient for distinctly elucidating the particular binding mechanisms of ligands to polynucleotides.

We examined the new guanidino-aryl compounds (**GA**) and porphyrin-guanidino **PoGU** bound to the G-quadruplex structure. Despite negligible thermal stabilization of the G-quadruplex structure, **GA1**-**GA4** showed significant fluorescence increase upon strong binding to the Tel22 G-quadruplex structure. The 1:1 stoichiometry complex is stabilised by a combination of π-π interactions of aryl groups and interactions from the guanidino group, where the interactions of the GA ligand’s guanidino cation with the oligonucleotide, either via hydrogen bonding or nonspecific electrostatic interactions, predominates in stabilising the complex. Porphyrin-based guanidino derivative **PoGU** exhibited a distinct binding profile, where titration with Tel22 resulted in fluorescence quenching. Most probably, at a higher excess of oligonucleotide, a 1:2 stoichiometry complex is formed, with the porphyrin compound **PoGU** between two quadruplex units. This sandwich-type complex is stabilized by strong π-π interaction between the large porphyrin core and G-tetrads.

Guanidino-aryl compounds **GA1**-**GA4** and **PoGU** were examined for antimetabolic activity against HeLa (human cervical adenocarcinoma) and MDCK1 (normal epithelial cell line). Guanidino-fluorene **GA3** exhibited moderate antimetabolic efficacy against the HeLa cell line, but no selectivity against the healthy cell line.

These results showed a strong impact of aromatic structure to the binding properties of aryl-guanidino compounds, which is relevant to the rational design of novel guanidino derivatives targeting nucleic acid structures for therapeutic or diagnostic purposes.

## Figures and Tables

**Table 1:** The Δ*T_m_* values ^a^ (°C) of studied *ds*-polynucleotides upon addition of guanidine compounds **GA1**-**GA4**, at ratio *r* = 0.3 ^b^.

	Δ*Tm* ^a^/°C		
	*ct*-DNA	poly rA-poly rU	poly dAdT-poly dAdT
**GA1**	+1.1	+1.3	+0.9
**GA2**	0	+0.9	0
**GA3**	+0.5	−1.0	0
**GA4**	+1.8	0	0

^a^ Δ*T_m_* = *T_m_* (complex) − *T_m_* (polynucleotide); error in Δ*T_m_*: ±0.5 °C; ^b^ *r* = [compound]/[polynucleotide].

**Table 2:** Stability constants (log *K_s_*) for fixed ratios *n*_[bound dye]/[polynucleotide]_ = 0.2 ^a,b^ of complexes of **GA1**-**GA4** compounds with *ds*-polynucleotides at pH = 7.0 (buffer sodium cacodylate, *I* = 0.05 mol dm^−3^) calculated from fluorimetric titration data.

	log *Ks* ^a^			
	*ct*-DNA	poly dAdT-poly dAdT	poly dGdC-poly dGdC	poly rA-poly rU
**GA1**	5.56	6.27	5.34	5–6 ^c^
**GA2**	5.46	5–6 ^c^	5–6 ^c^	5–6 ^c^
**GA3**	5–6 ^c^	5–6 ^c^	^d^	4–5 ^c^
**GA4**	5–6 ^c^	4.17	^d^	^d^

^a,b^ Processing of titration data by means of Scatchard equation [40,41] gave stability constants *Ks*; all *Ks* values were recalculated with a fixed value *n* = 0.2 for comparison reasons; correlation coefficients were >0.99 for all calculated *Ks.* ^c^ Small total emission change and/or high divergences of fluorescence intensities enabled only the estimation of the stability constant. ^d^ Small fluorescence change and/or linear fluorescence change disabled collecting enough data for the calculation of the stability constant.

**Table 3:** Stability constants (log *K*) ^a^ of **GA1**-**GA4** and **PoGU**-Tel22 complexes at pH = 7.0 (buffer sodium cacodylate, *I* = 0.1 mol dm^−3^) calculated from fluorimetric titrations data.

Compound	log *K*_1_; log*K*_2_ ^b^
**GA1**	5.61 ± 0.02
**GA2**	5.26 ± 0.13
**GA3**	5.78 ± 0.08
**GA4**	5.36 ± 0.07
**PoGU23**	7.23 ± 0.49; 5.07 ± 0.26

^a^ Stability constant log *K* and stoichiometry calculated by processing the titration data by the SPECFIT program [54,55,56]. ^b^ *K*_1_ and *K*_2_ refer to the equilibria L + G ⇆ LG and LG + G ⇆ LG_2_ (L = ligand, G = G-quadruplex), respectively; *K*_2_ stability constant was determined for **PoGU**.

## Data Availability

The original contributions presented in this study are included in the article/supplementary material. Further inquiries can be directed to the corresponding authors.

## Supplementary Materials

The following supporting information can be downloaded at https://www.mdpi.com/article/10.3390/molecules30183682/s1: The supporting information contain UV-Vis data of **GA1**-**GA4**, groove widths and depths for selected nucleic acid conformations, ∆*T_m_* values and melting curves of polynucleotides and Tell22 complexes with **GA1**-**GA4**, fluorimetric titration data, CD titration data, dose-response profiles for **GA1**-**GA4** and **PoGU** in tumour and healthy cell lines, 1H/13C NMR and mass spectrum data of **GA1**-**GA4.** References [2,23,28,40,41] are cited in the Supplementary Materials.
