# Supplementary material for: Guanidino-Aryl Derivatives: Binding to DNA, RNA and G-Quadruplex Structure and Antimetabolic Activity
Source: Molecules. 2025 Sep 10;30(18):3682. doi: 10.3390/molecules30183682 (PMC12472303; doi:10.3390/molecules30183682)
Supplement: Supplementary file 1 [file molecules-30-03682-s001.zip › molecules-3787741-supplementary.pdf]

SUPPORTING INFORMATION

Guanidino-aryl derivatives: binding to DNA, RNA and G-quadruplex structure and antimetabolic activity

Davor Margetić [1]*, Petra Jadrijević-Mladar [2], Anamaria Brozovic [2] and Lidija-Marija Tumir [1]*

[1] Division of Organic Chemistry & Biochemistry, Ruđer Bošković Institute, Zagreb, Croatia; Davor.Margetic@irb.hr (DM), tumir@irb.hr (LMT)
[2] Division of Molecular Biology, Ruđer Bošković Institute, Zagreb, Croatia; Anamaria.Brozovic@irb.hr (AB), Petra.Jadrijevic-Mladar@irb.hr (PJM)
***Correspondence:** tumir@irb.hr (LMT); Davor.Margetic@irb.hr (DM) Tel.: (+385 1 457 1220, LMT)

Content

1. Spectroscopic properties of **GA1-GA4**
2. Interactions of **GA1-GA4** and **PoGU** with DNA/RNA and Tel22
3. Biological Activity
4. NMR and mass spectra of **GA1-GA4**

1. Spectroscopic properties of compounds in water media

Table S1. Electronic absorption data of **guanidino-aryl** compounds **GA1-GA4** and **PoGU** [1] (sodium cacodylate buffer, I = 0.05 mol dm^{-3}, pH = 7)

	λ_{max} / nm	ε / mmol^{-1} cm^2
**GA1**	253	7800
**GA2**	276	291
**GA3**	267	28800
**GA4**	276	3700
**PoGU** [1]	424	84900

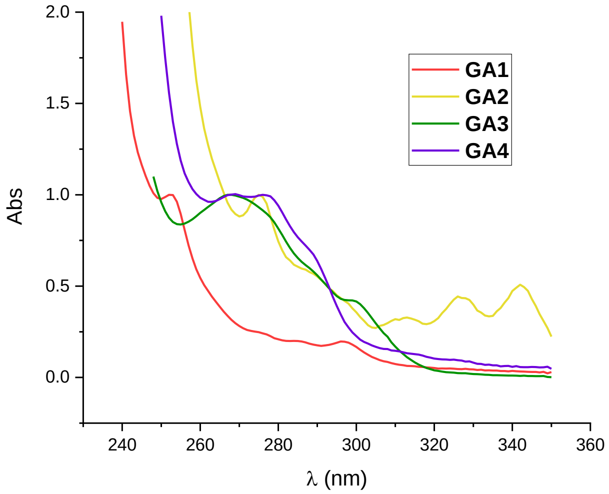

Figure S1. The UV/Vis spectra of the **GA1-GA4**: absorbencies were normalised at λmax for each compound (c =2-4×10^{-5} mol dm^{-3}, Na-cacodylate buffer, I = 0.05 mol dm^{-3}, pH = 7.0) (it is important to note that absorption bands and maxima of compounds below 240 nm could not be quantified due to strong absorption of UV light by DMSO originating from stock solution)

2. Interactions of GA1-GA4 with DNA/RNA and G-quadruplex

Table S2. Groove widths and depths for selected nucleic acid conformations[2],[3].

Structure type	Groove width [Å]		Groove depth [Å]	
	major	minor	major	minor
[a] poly rA – poly rU	3.8	10.9	13.5	2.8
[b] B-DNA (e.g. *ct*-DNA)	11.7	5.7	8.5	7.5
[b] poly dGdC – poly dGdC	13.5	9.5	10.0	7.2
[b] poly dAdT – poly dAdT	11.2	6.3	8.5	7.5

[a] A-helical structure (e.g., A-DNA or RNA)

[b] B- helical structure (e.g., B-DNA)

3.1. Thermal melting results

Table S3. The ΔT_m values[a] (°C) of Tel22 G-quadruplex structure upon addition of guanidine compounds **GA1-GA4** and **PoGU**, at ratio *r* [compound] / [Tel22; expressed as oligonucleotide] = 4

	**ΔTm^{a}/ °C**
**GA1**	-0.8
**GA2**	0
**GA3**	0
**GA4**	+1.1
**PoGU**	0

[a]ΔT_m = T_m (complex) –T_m (Tel22); Error in ΔT_m : ±0.5°C;

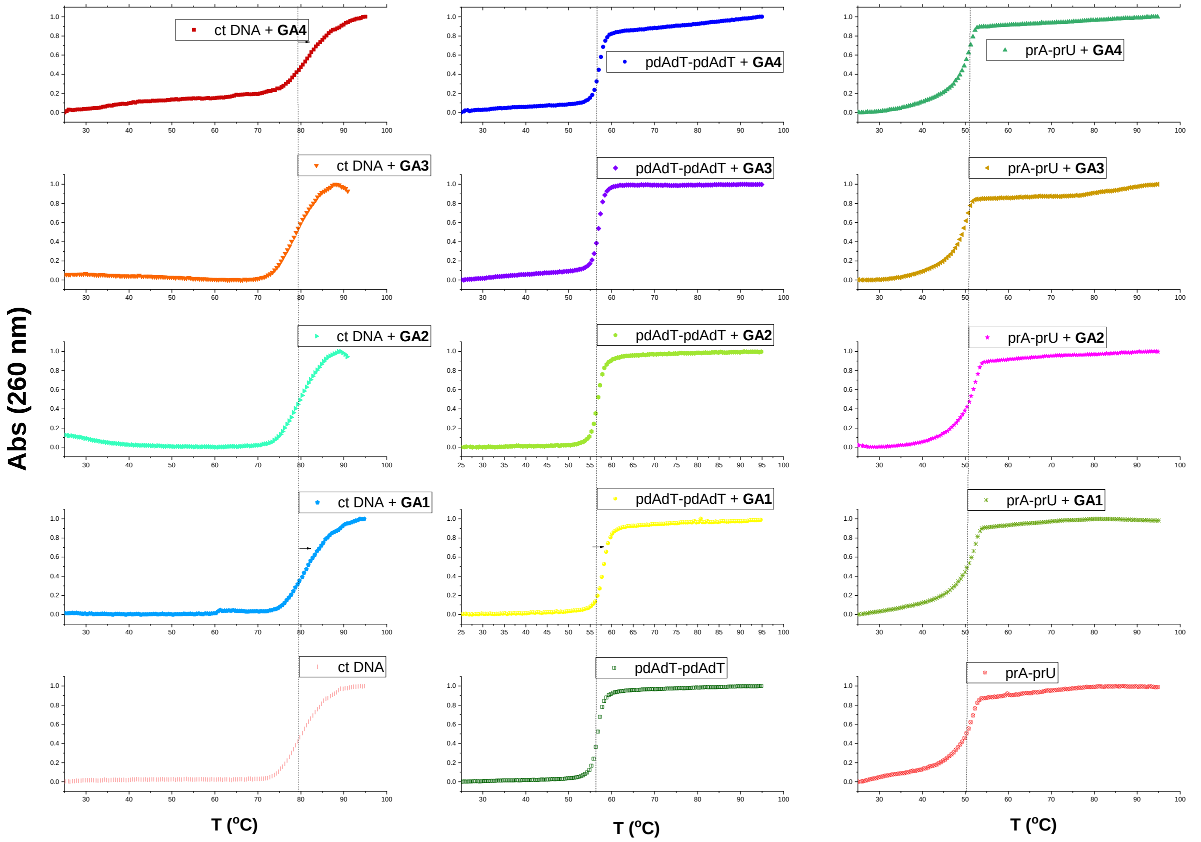

Figure S2. Melting curves of *ct*-DNA, poly dAdT-poly dAdT and poly rA-poly rU upon addition of **GA1-GA4** compounds (c (DNA or RNA) = 2 ×10^{-5} M; ratio r[compound] /[polynucleotide phosphate] = 0.3) at pH = 7.0 (sodium cacodylate buffer, I = 0.05 mol dm^{-3}).

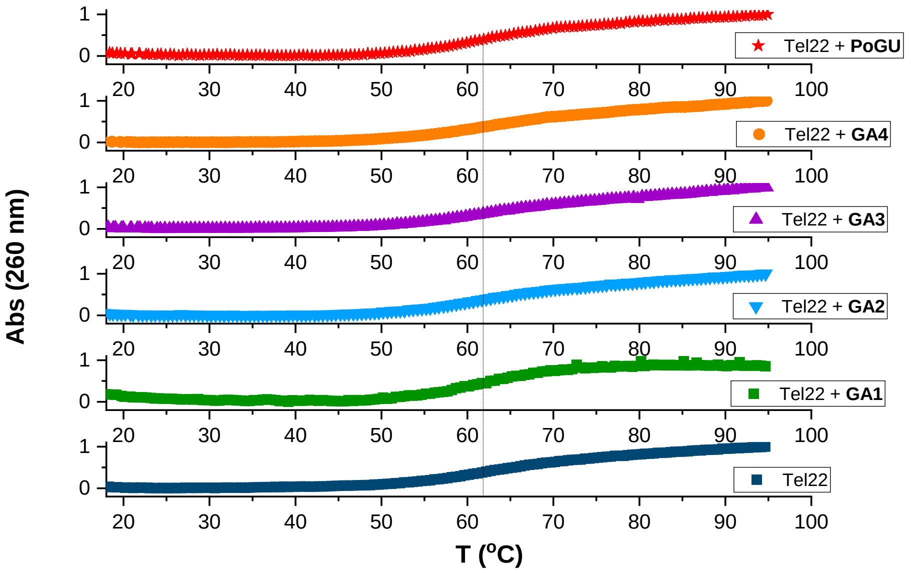

Figure S3. Melting curves of **Tel22** upon addition of **GA1-GA4** and **PoGU** compounds (c (Tel22)=1-2 ×10^{-6} M (expressed as oligonucleotide concentration); ratio r [compound] /[Tel22] = 4) at pH = 7.0 (sodium cacodylate buffer, I = 0.1 mol dm^{-3}).

3.2. Fluorimetric titrations with DNA/RNA

Titration data (GA compounds + polynucleotides) were processed by Scatchard equation [[4]5] to calculate stability constants and ratio $n_{\text{[bound compound] / [polynucleotide]}}$

Nonlinear Scatchard equation:

$$I = I_0 + ((I_{lim} - I_0) / (2\times c)) \times (c + n \times c_s + 1 / K_a - ((c + n \times c_s + 1 / K_a)^2 - 4 \times c \times n \times c_s)^{1/2})$$

where I is fluorescence intensity, while I_0 and I_{lim} denote fluorescence intensities of free and fully complexed ligand; c is concentration of free ligand; c_s is concentration of polynucleotide; n is ratio[bound ligand]/[polynucleotide]; K_a is complex association constant.

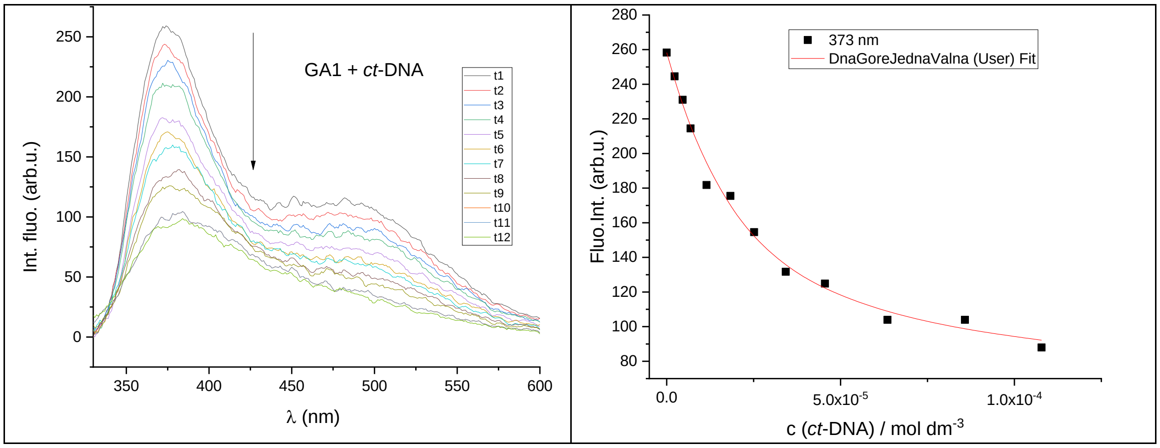

Figure S4. Left: Fluorimetric titration of **GA1**, λ_{exc} =300 nm, c =2.5 × 10^{-6} mol dm^{-3} with *ct*-DNA, Right: Experimental (●) and calculated (—) (fix n=0.2) fluorescence intensities of **GA1** at λ_{em} = 373 nm upon addition of DNA (pH = 7.0, Na cacodylate buffer, I = 0.05 mol dm^{-3}).

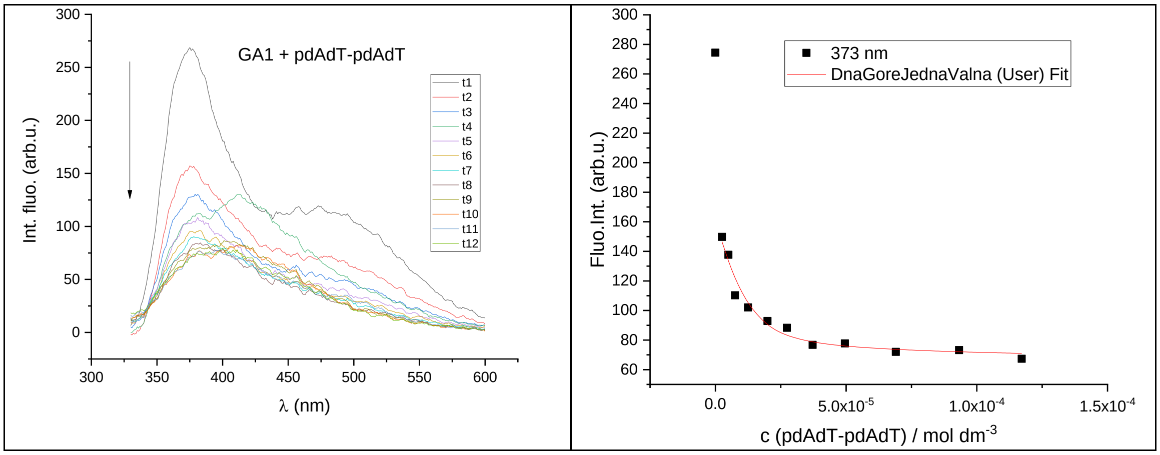

Figure S5. Left: Fluorimetric titration of **GA1**, λ_{exc} =300 nm, c =2.5 × 10^{-6} mol dm^{-3} with pdAdT-pdAdT, Right: Experimental (●) and calculated (—) (fix n=0.2) fluorescence intensities of **GA1** at λ_{em} = 373 nm upon addition of DNA (pH = 7.0, Na cacodylate buffer, I = 0.05 mol dm^{-3}).

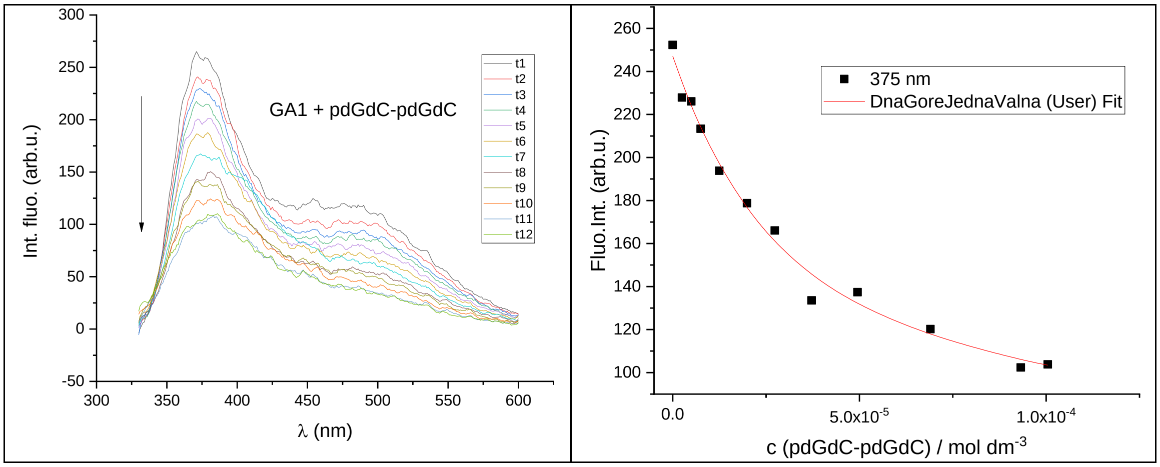

Figure S6. Left: Fluorimetric titration of **GA1**, λ_{exc} =300 nm, c =2.5 × 10^{-6} mol dm^{-3} with pdGdC-pdGdC, Right: Experimental (●) and calculated (—) (fix n=0.2) fluorescence intensities of **GA1** at λ_{em} = 375 nm upon addition of DNA (pH = 7.0, Na cacodylate buffer, I = 0.05 mol dm^{-3}).

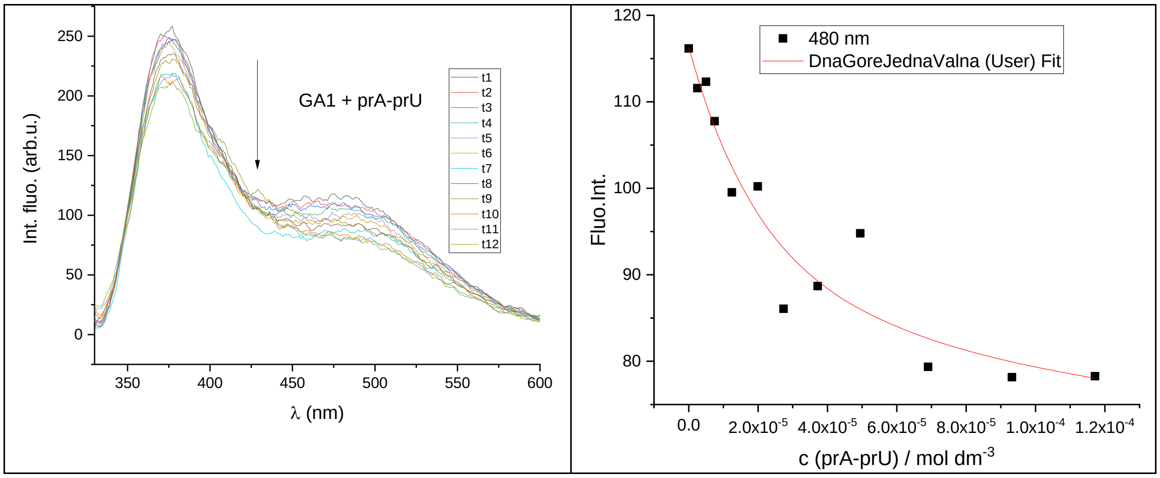

Figure S7. Left: Fluorimetric titration of **GA1**, λ_{exc} = 300 nm, c =2.5 × 10^{-6} mol dm^{-3} with prA-prU, Right: Experimental (●) and calculated (—) (fix n=0.2) fluorescence intensities of **GA1** at λ_{em} = 480 nm upon addition of RNA (pH = 7.0, Na cacodylate buffer, I = 0.05 mol dm^{-3}).

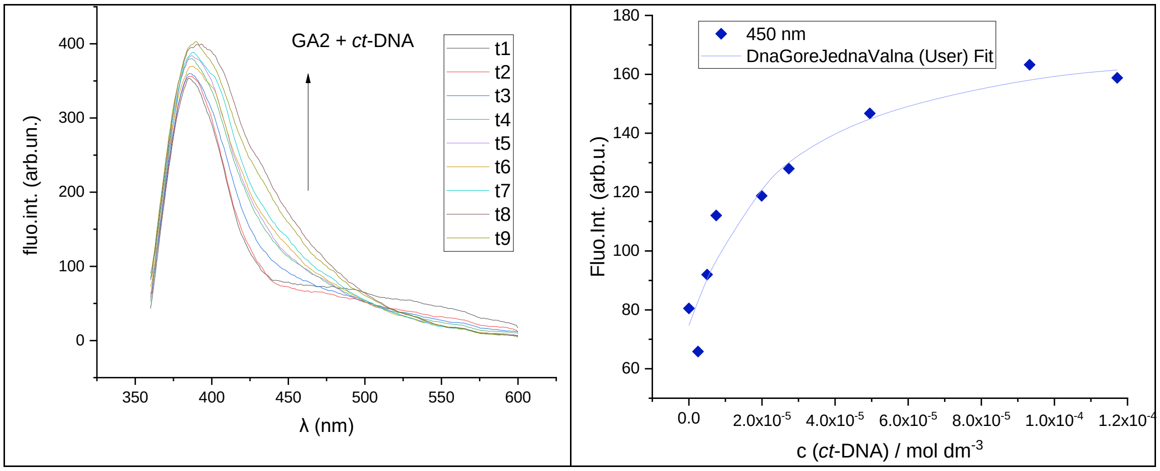

Figure S8. Left: Fluorimetric titration of **GA2**, λ_{exc} =325 nm, c =2.5 × 10^{-6} mol dm^{-3} with *ct*-DNA, Right: Experimental (●) and calculated (—) (fix n=0.2) fluorescence intensities of **GA2** at λ_{em} = 450 nm upon addition of DNA (pH = 7.0, Na cacodylate buffer, I = 0.05 mol dm^{-3}).

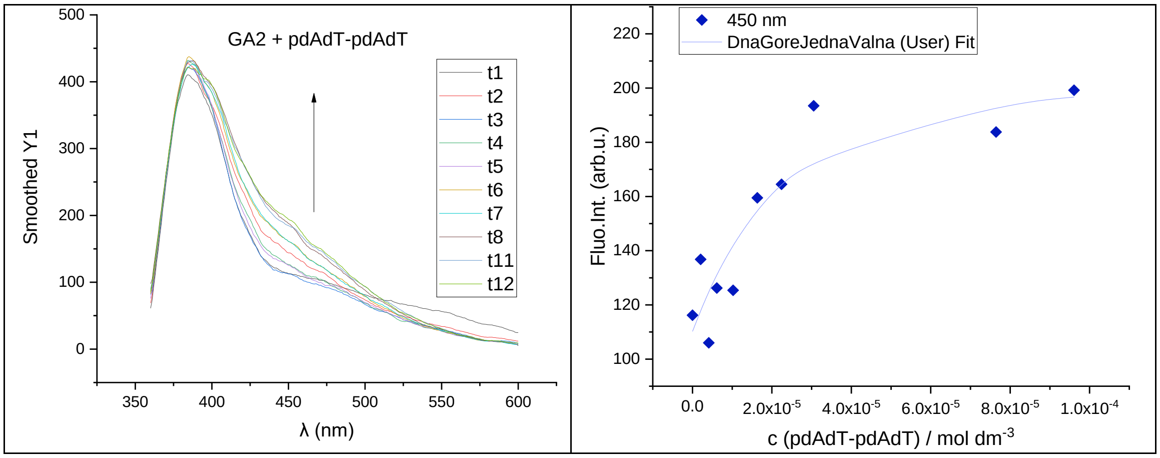

Figure S9. Left: Fluorimetric titration of **GA2**, λ_{exc} =325 nm, c =2.5 × 10^{-6} mol dm^{-3} with pdAdT-pdAdT, Right: Experimental (●) and calculated (—) (fix n=0.2) fluorescence intensities of **GA2** at λ_{em} = 450 nm upon addition of DNA (pH = 7.0, Na cacodylate buffer, I = 0.05 mol dm^{-3}).

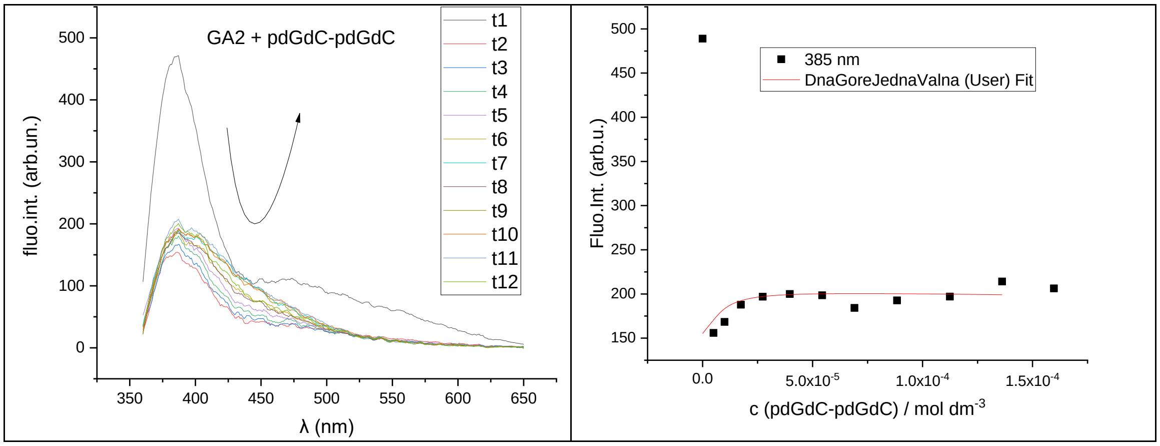

Figure S10. Left: Fluorimetric titration of **GA2**, λ_{exc} =325 nm, c =2.5 × 10^{-6} mol dm^{-3} with pdGdC-pdGdC, Right: Experimental (●) and calculated (—) (fix n=0.2) fluorescence intensities of **GA2** at λ_{em} = 385 nm upon addition of DNA (pH = 7.0, Na cacodylate buffer, I = 0.05 mol dm^{-3}).

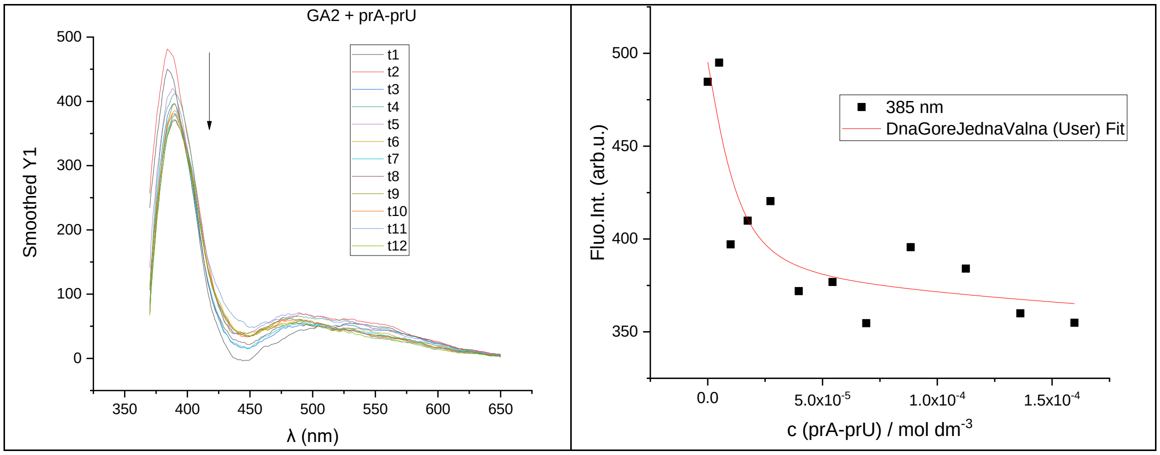

Figure S11. Left: Fluorimetric titration of **GA2**, λ_{exc} =325 nm, c =2.5 × 10^{-6} mol dm^{-3} with prA-prU, Right: Experimental (●) and calculated (—) (fix n=0.2) fluorescence intensities of **GA2** at λ_{em} = 385 nm upon addition of RNA (pH = 7.0, Na cacodylate buffer, I = 0.05 mol dm^{-3}).

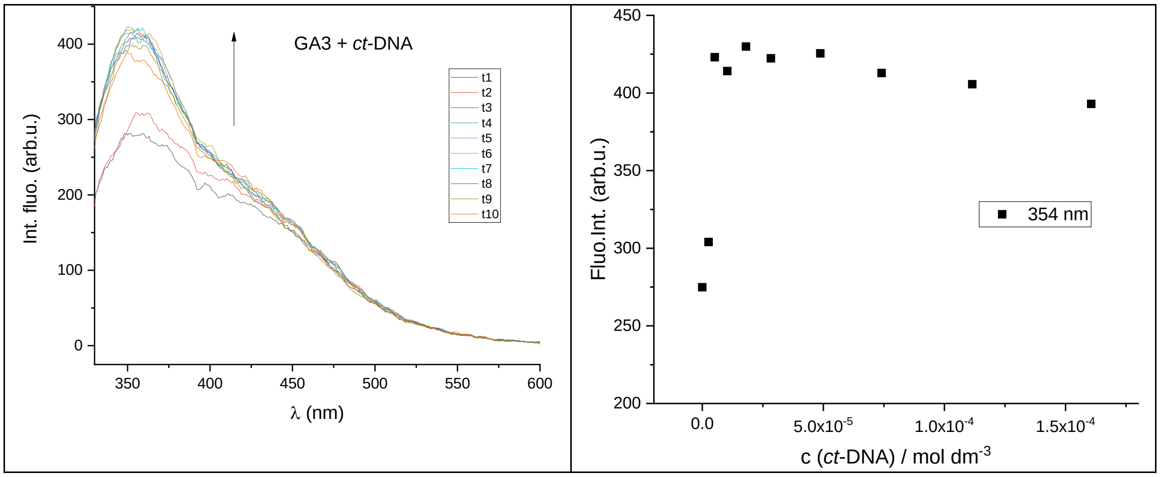

Figure S12. Left: Fluorimetric titration of **GA3**, λ_{exc} =300 nm, c =2.5 × 10^{-6} mol dm^{-3} with *ct*-DNA, Right: Experimental (●) fluorescence intensities of **GA3** at λ_{em} = 354 nm upon addition of DNA (pH = 7.0, Na cacodylate buffer, I = 0.05 mol dm^{-3}).

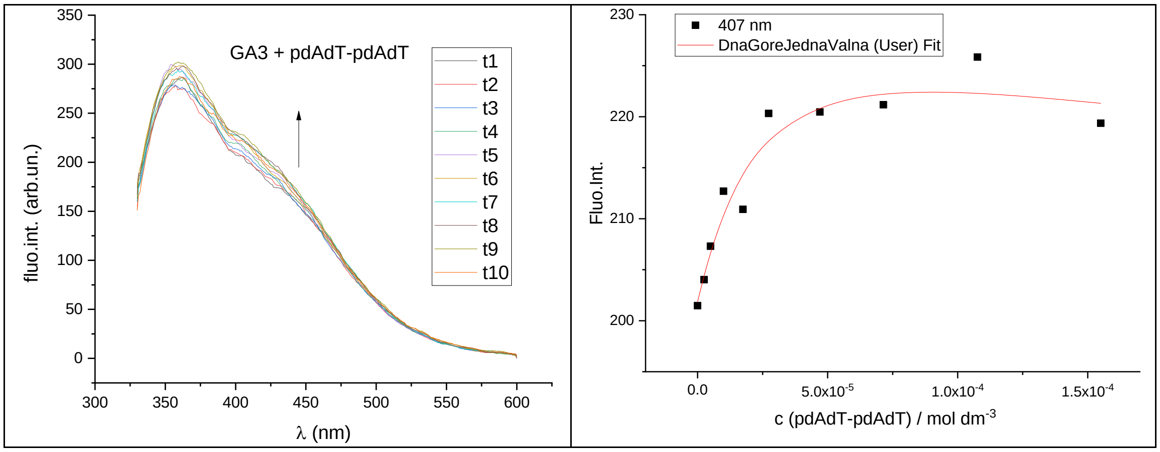

Figure S13. Left: Fluorimetric titration of **GA3**, λ_{exc}=300 nm, c =2.5 × 10^{-6} mol dm^{-3} with pdAdT-pdAdT, Right: Experimental (●) and calculated (—) (fix n=0.2) fluorescence intensities of **GA3** at λ_{em} = 373 nm upon addition of DNA (pH = 7.0, Na cacodylate buffer, I = 0.05 mol dm^{-3}).

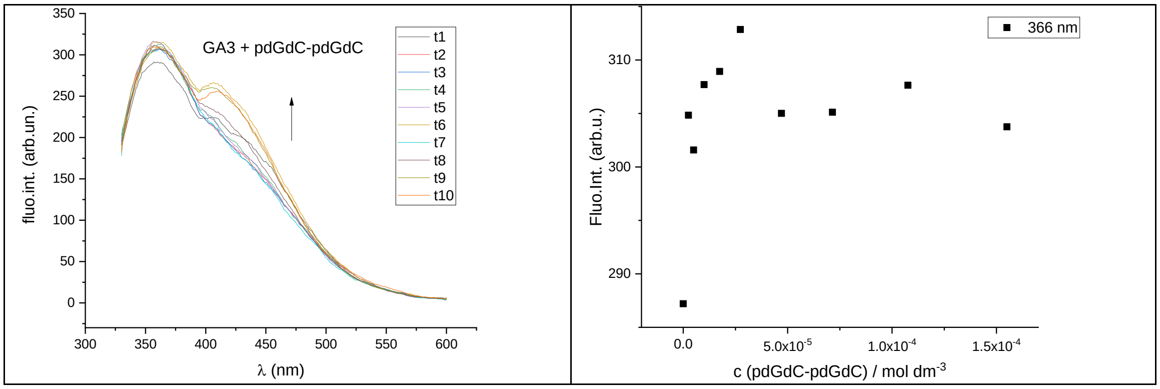

Figure S14. Left: Fluorimetric titration of **GA3**, λ_{exc}=300 nm, c =2.5 × 10^{-6} mol dm^{-3} with pdGdC-pdGdC, Right: Experimental (●) fluorescence intensities of **GA3** at λ_{em} = 366 nm upon addition of DNA (pH = 7.0, Na cacodylate buffer, I = 0.05 mol dm^{-3}).

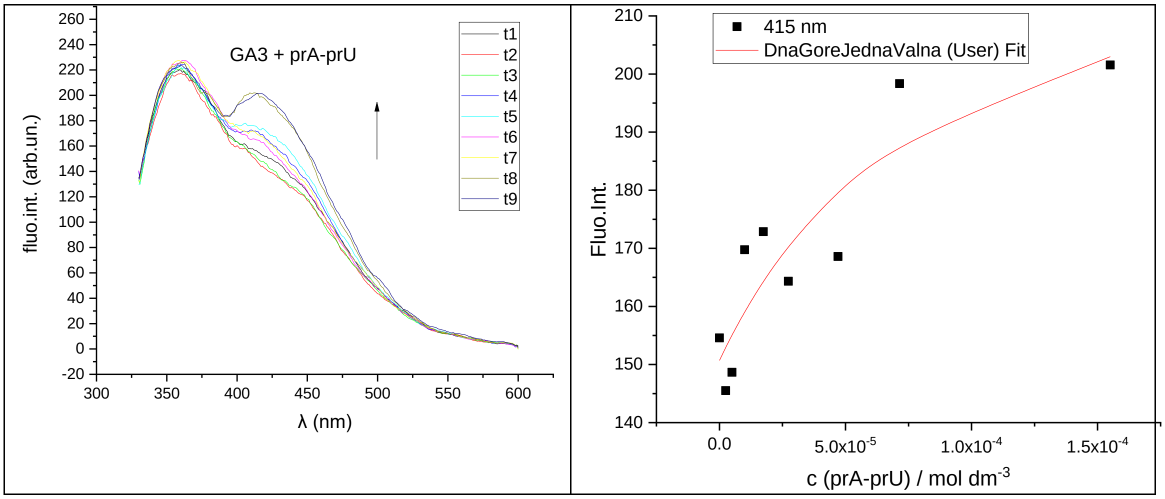

Figure S15. Left: Fluorimetric titration of **GA3**, λ_{exc}=300 nm, c =2.5 × 10^{-6} mol dm^{-3} with prA-prU, Right: Experimental (●) and calculated (—) (fix n=0.2) fluorescence intensities of **GA3** at λ_{em} = 415 nm upon addition of RNA (pH = 7.0, Na cacodylate buffer, I = 0.05 mol dm^{-3}).

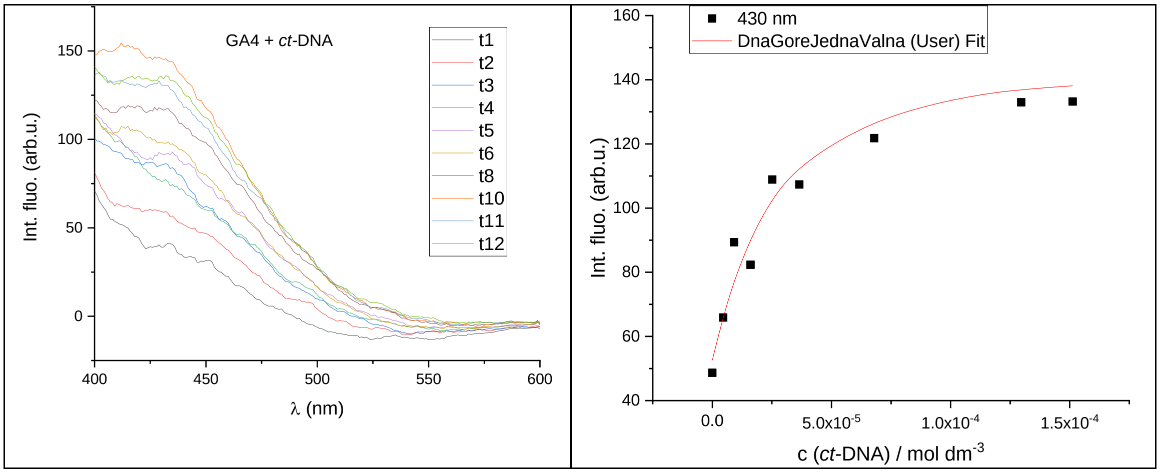

Figure S16. Left: Fluorimetric titration of **GA4**, λ_{exc} =300 nm, c =2.5 × 10^{-6} mol dm^{-3} with *ct*-DNA, Right: Experimental (●) and calculated (—) (fix n=0.2) fluorescence intensities of **GA4** at λ_{em} = 373 nm upon addition of DNA (pH = 7.0, Na cacodylate buffer, I = 0.05 mol dm^{-3}).

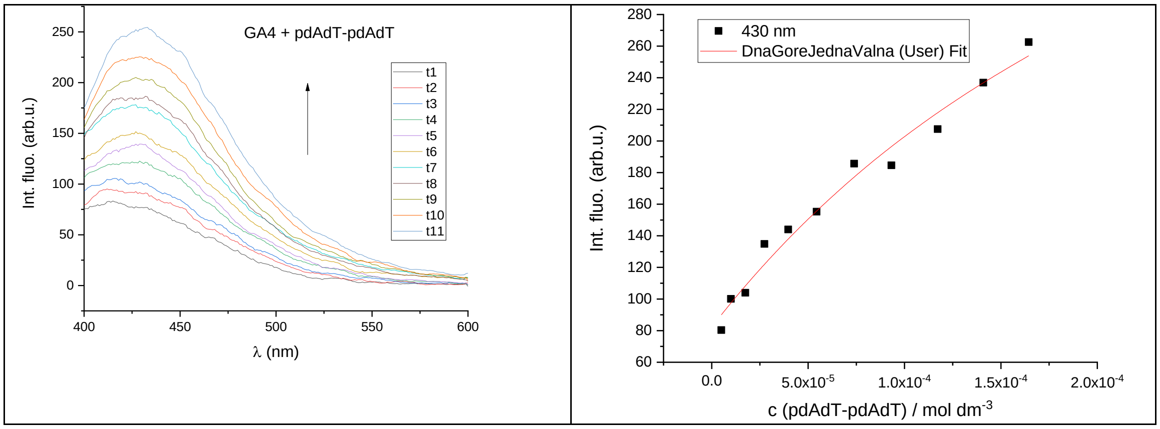

Figure S17. Left: Fluorimetric titration of **GA4**, λ_{exc} =300 nm, c =2.5 × 10^{-6} mol dm^{-3} with pdAdT-pdAdT, Right: Experimental (●) and calculated (—) (fix n=0.2) fluorescence intensities of **GA4** at λ_{em} = 373 nm upon addition of DNA (pH = 7.0, Na cacodylate buffer, I = 0.05 mol dm^{-3}).

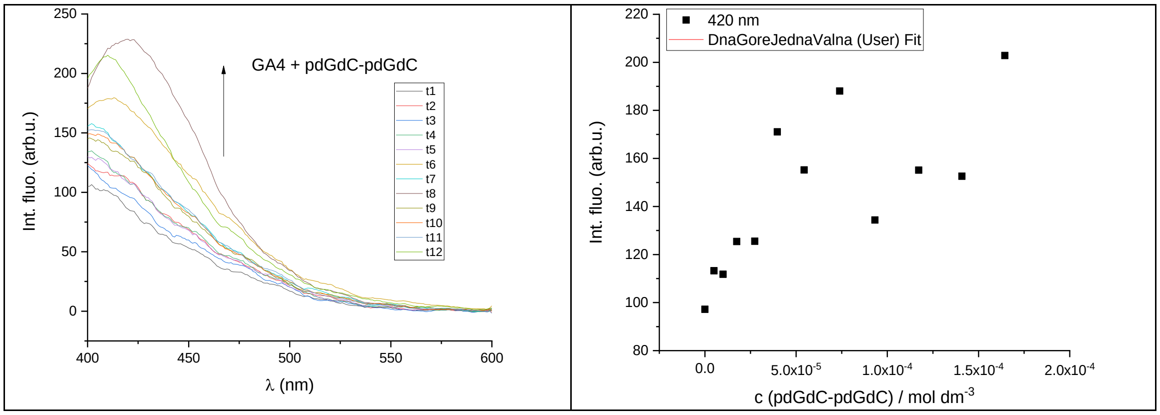

Figure S18. Left: Fluorimetric titration of **GA4**, λ_{exc} =300 nm, c =2.5 × 10^{-6} mol dm^{-3} with pdGdC-pdGdC, Right: Experimental (●) fluorescence intensities of **GA4** at λ_{em} = 375 nm upon addition of DNA (pH = 7.0, Na cacodylate buffer, I = 0.05 mol dm^{-3}).

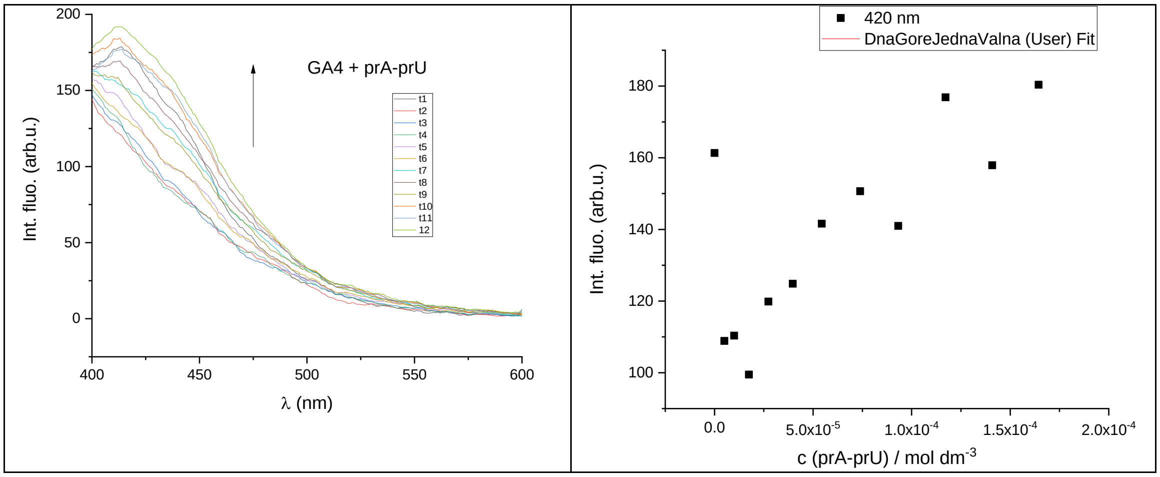

Figure S19. Left: Fluorimetric titration of **GA4**, λ_{exc} =300 nm, c =2.5 × 10^{-6} mol dm^{-3} with prA-prU, Right: Experimental (●) fluorescence intensities of **GA4** at λ_{em} = 420 nm upon addition of RNA (pH = 7.0, Na cacodylate buffer, I = 0.05 mol dm^{-3}).

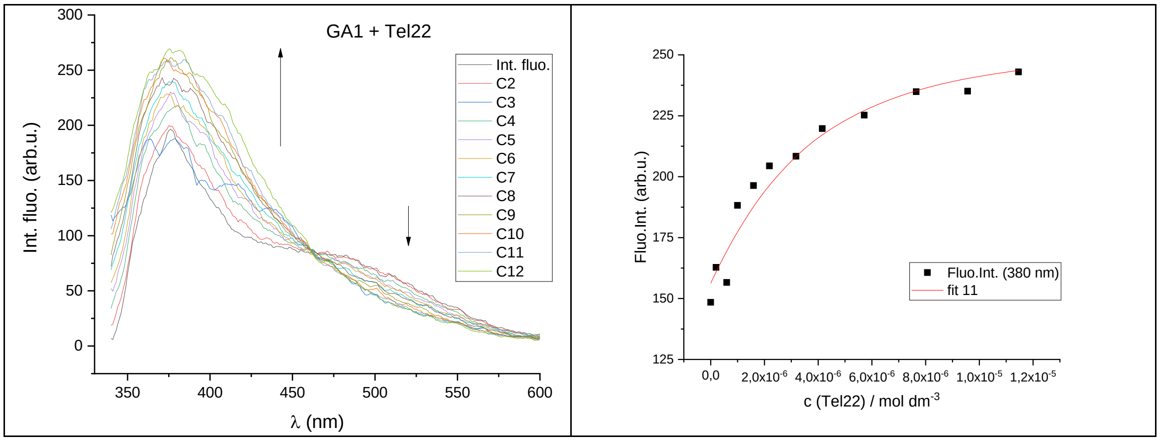

Figure S20. Left: Fluorimetric titration of **GA1**, λ_{exc} =300 nm, c =2.5 × 10^{-6} mol dm^{-3} with Tel22, Right: Experimental (●) and calculated (—) fluorescence intensities of **GA1-Tel22** complex at λ_{em} = 380 nm (pH = 7.0, Na cacodylate buffer, I = 0.1 mol dm^{-3})

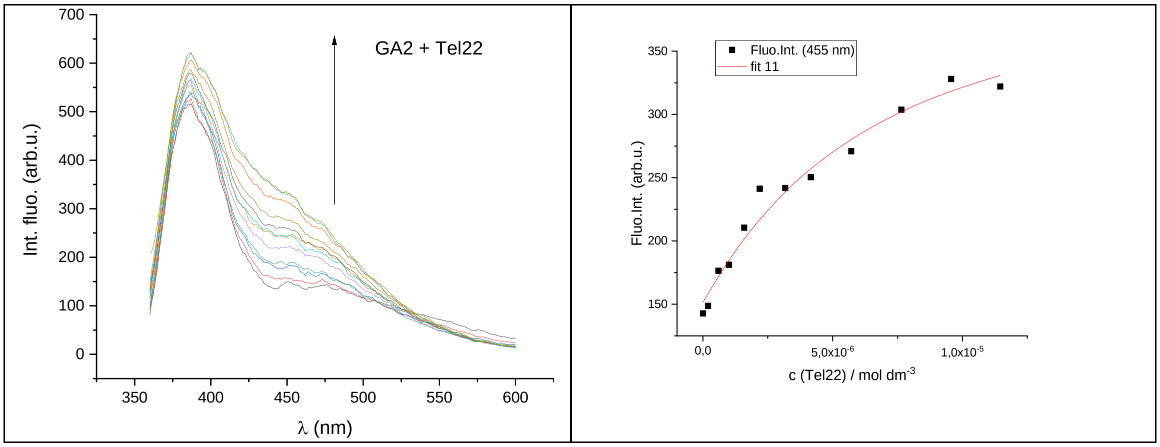

Figure S21. Left: Fluorimetric titration of **GA2**, λ_{exc} =300 nm, c =2.5 × 10^{-6} mol dm^{-3} with Tel22, Right: Experimental (●) and calculated (—) fluorescence intensities of **GA2-Tel22** complex at λ_{em} = 455 nm (pH = 7.0, Na cacodylate buffer, I = 0.1 mol dm^{-3})

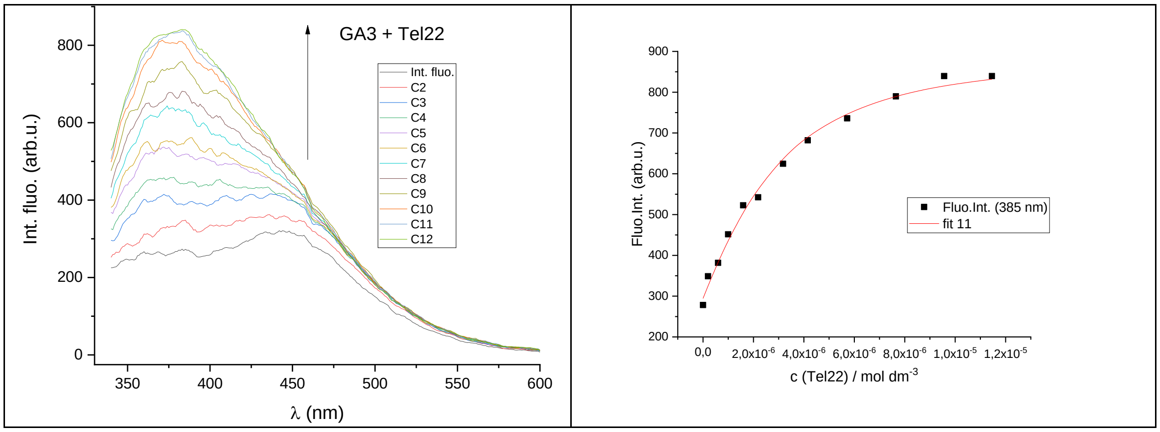

Figure S22. Left: Fluorimetric titration of **GA3**, λ_{exc} =300 nm, c =2.5 × 10^{-6} mol dm^{-3} with Tel22, Right: Experimental (●) and calculated (—) fluorescence intensities of **GA3-Tel22** complex at λ_{em} = 385 nm (pH = 7.0, Na cacodylate buffer, I = 0.1 mol dm^{-3})

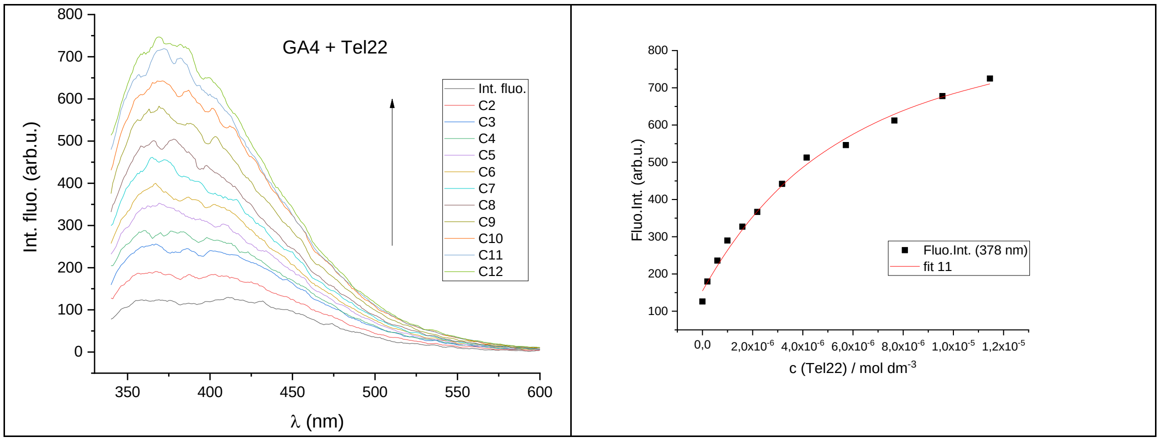

Figure S23. Left: Fluorimetric titration of **GA4**, λ_{exc} =300 nm, c =2.5 × 10^{-6} mol dm^{-3} with Tel22, Right: Experimental (●) and calculated (—) fluorescence intensities of **GA4-Tel22** complex at λ_{em} = 378 nm (pH = 7.0, Na cacodylate buffer, I = 0.1 mol dm^{-3})

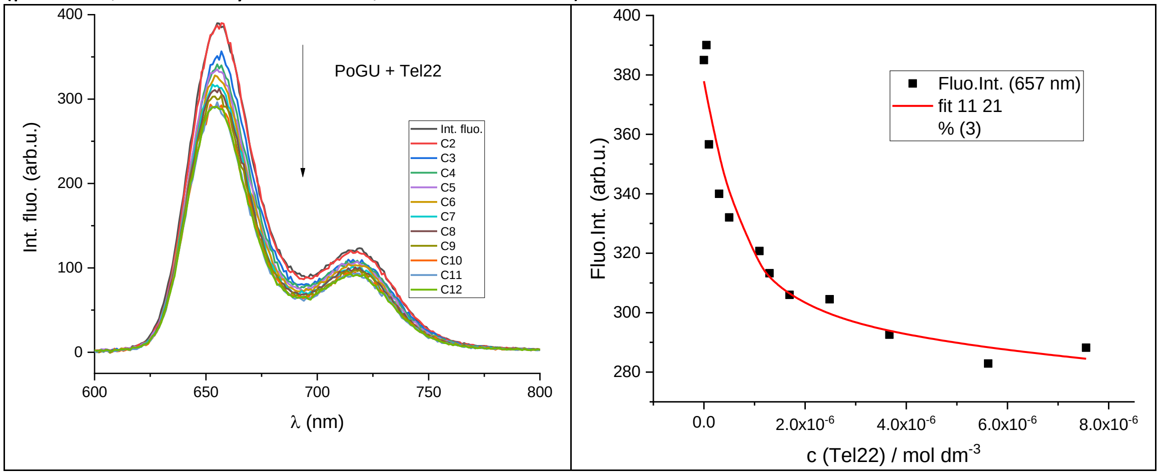

Figure S24. Left: Fluorimetric titration of **PoGU**, λ_{exc} =420 nm, c =1 × 10^{-6} mol dm^{-3} with Tel22, Right: Experimental (●) and calculated (—) fluorescence intensities of **PoGU -Tel22** complex at λ_{em} = 657 nm (pH = 7.0, Na cacodylate buffer, I = 0.1 mol dm^{-3})

3.3. Circular dichroism (CD) titrations with DNA/RNA and Tel22

CD spectroscopy was chosen to monitor conformational changes of polynucleotide secondary structure induced by small molecule binding. Compounds **GA1-GA4** are achiral and consequently have no intrinsic CD spectrum.

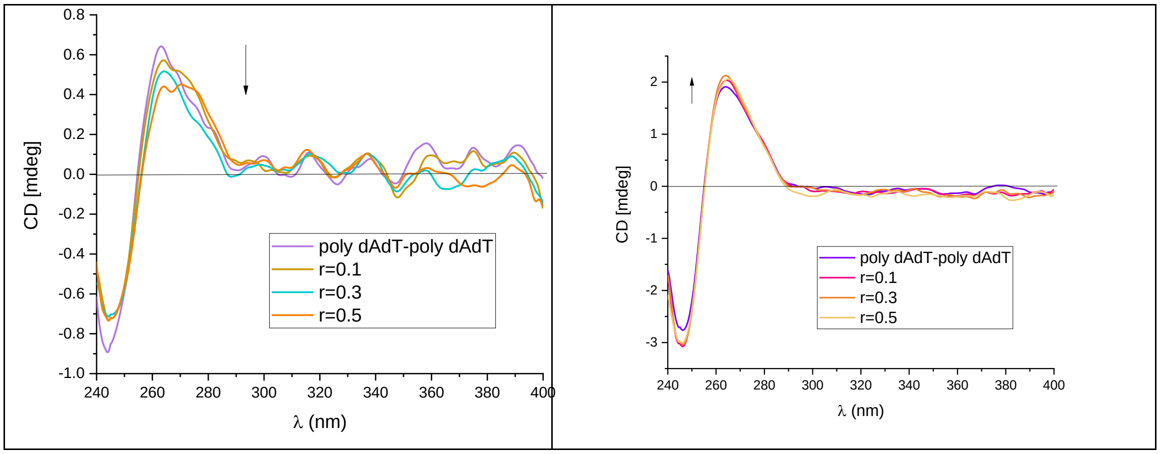

Figure S25. Changes in the CD spectrum of poly dAdT-poly dAdT (c(DNA) = 3 × 10^{-5} mol dm^{-3}) upon addition of **GA1** (left) and **GA2** (right) at different molar ratios r = [compound] / [polynucleotide], pH = 7.0, sodium cacodylate buffer, I =0.05 mol dm^{-3}

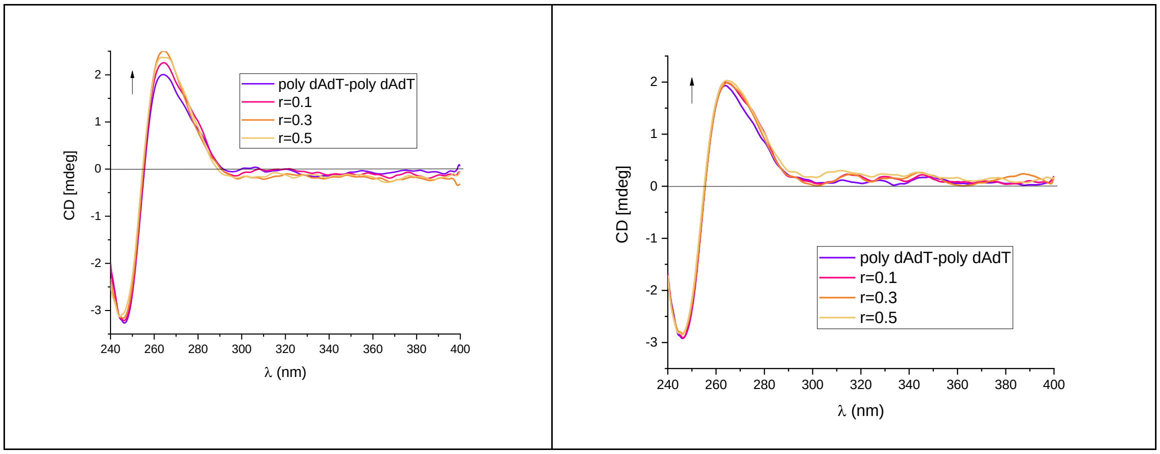

Figure S26. Changes in the CD spectrum of poly dAdT-poly dAdT (c(DNA) = 3 × 10^{-5} mol dm^{-3}) upon addition of **GA3** (left) and **GA4** (right) at different molar ratios r = [compound] / [polynucleotide], pH = 7.0, sodium cacodylate buffer, I =0.05 mol dm^{-3}

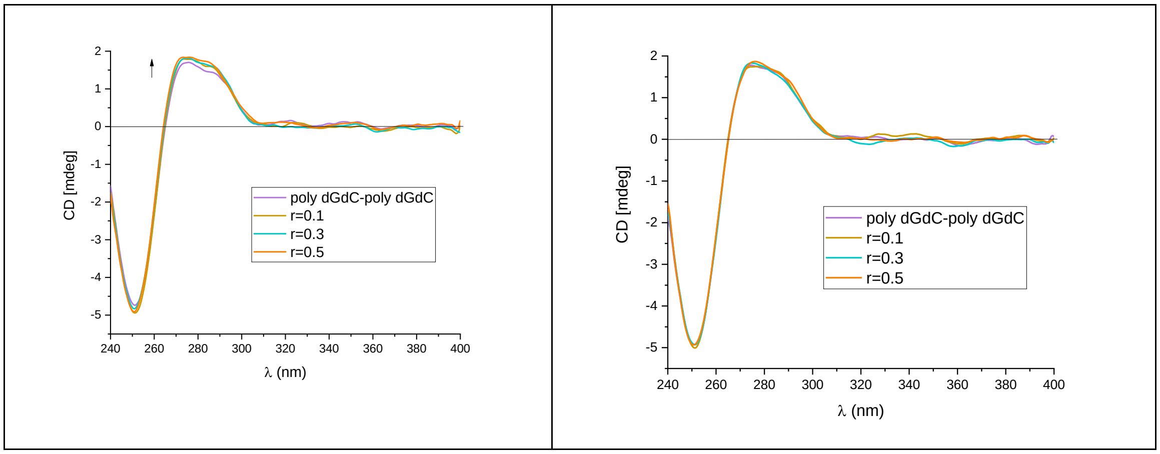

Figure S27. Changes in the CD spectrum of poly dGdC-poly dGdC (c(DNA) = 3 $\times$ 10^{-5} mol dm^{-3}) upon addition of **GA1** (left) and **GA2** (right) at different molar ratios r = [compound] / [polynucleotide], pH = 7.0, sodium cacodylate buffer, I =0.05 mol dm^{-3}

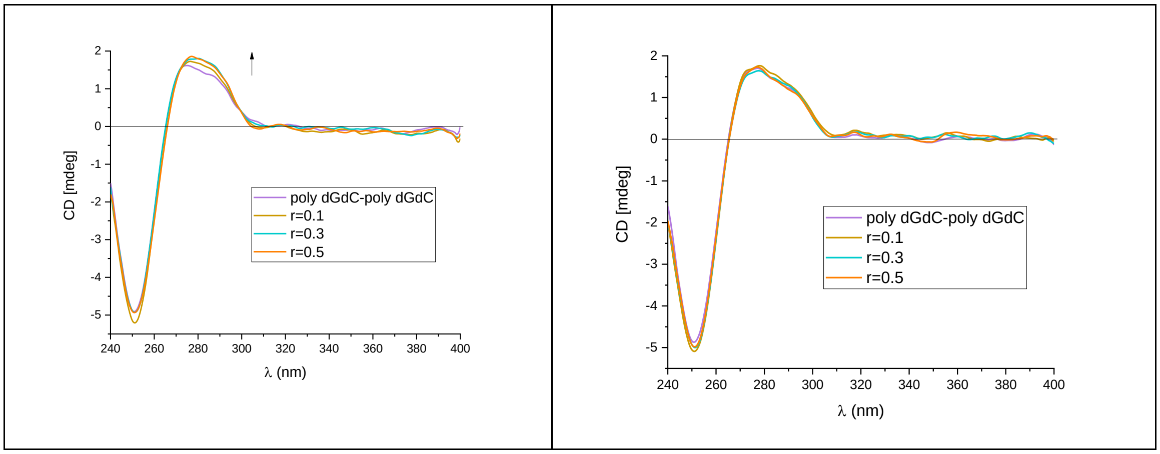

Figure S28. Changes in the CD spectrum of poly dGdC-poly dGdC (c(DNA) = 3 $\times$ 10^{-5} mol dm^{-3}) upon addition of **GA3** (left) and **GA4** (right) at different molar ratios r = [compound] / [polynucleotide], pH = 7.0, sodium cacodylate buffer, I =0.05 mol dm^{-3}

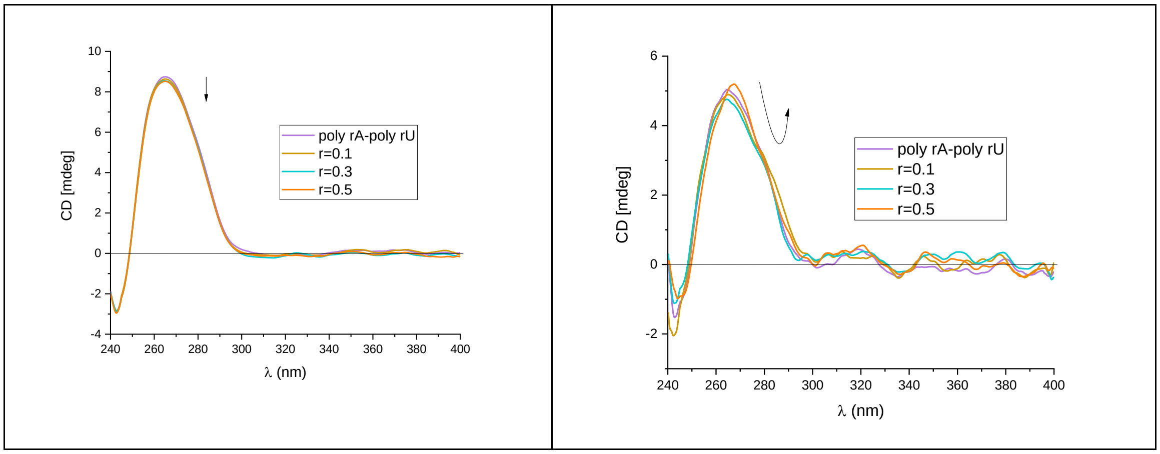

Figure S29. Changes in the CD spectrum of poly rA-poly rU (c(RNA) = 3 × 10^{-5} mol dm^{-3}) upon addition of **GA1** (left) and **GA2** (right) at different molar ratios r = [compound] / [polynucleotide], pH = 7.0, sodium cacodylate buffer, I =0.05 mol dm^{-3}

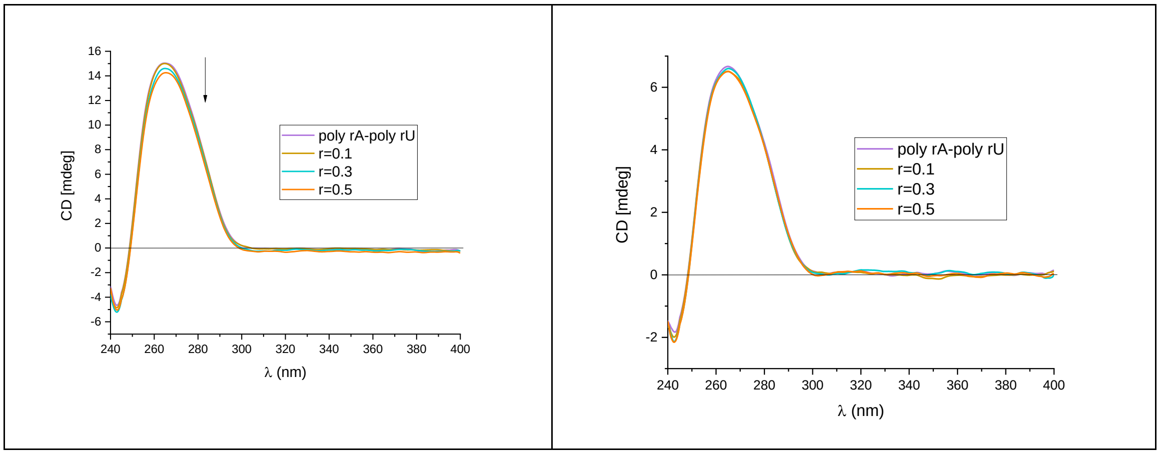

Figure S30. Changes in the CD spectrum of poly rA-poly rU (c(RNA) = 3 × 10^{-5} mol dm^{-3}) upon addition of **GA3** (left) and **GA4** (right) at different molar ratios r = [compound] / [polynucleotide], pH = 7.0, sodium cacodylate buffer, I =0.05 mol dm^{-3}

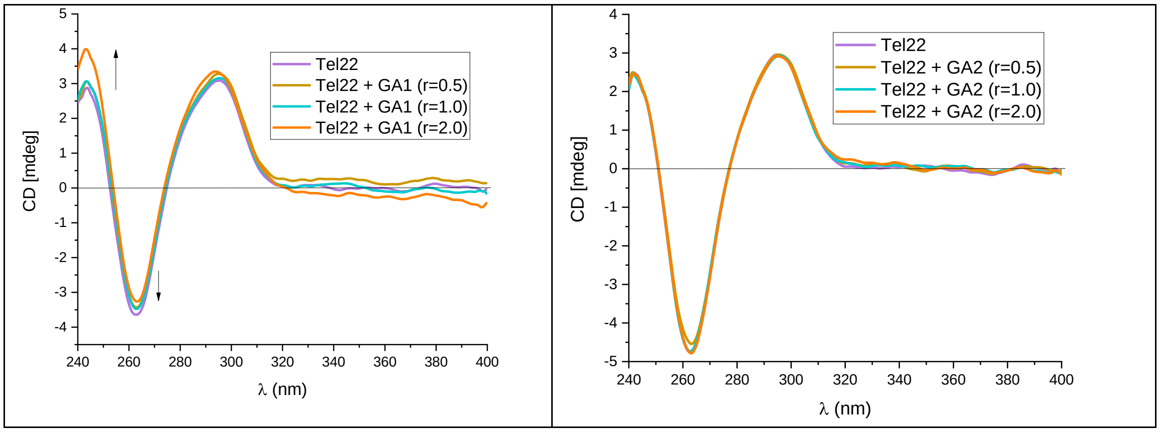

Figure S31. Changes in the CD spectrum of Tel22 (c =1 × 10^{-6} mol dm^{-3}) upon addition of **GA1** (left) and **GA2** (right) at different molar ratios r = [compound] / [polynucleotide], pH = 7.0, sodium cacodylate buffer, I =0.1 mol dm^{-3}

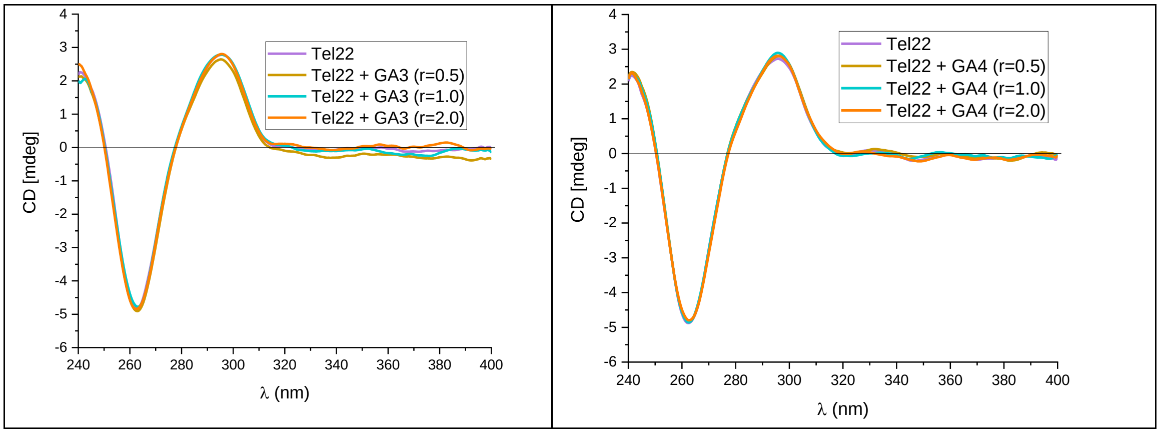

Figure S32. Changes in the CD spectrum of Tel22 (c =1 × 10^{-6} mol dm^{-3}) upon addition of **GA3** (left) and **GA4** (right) at different molar ratios r = [compound] / [polynucleotide], pH = 7.0, sodium cacodylate buffer, I =0.1 mol dm^{-3}

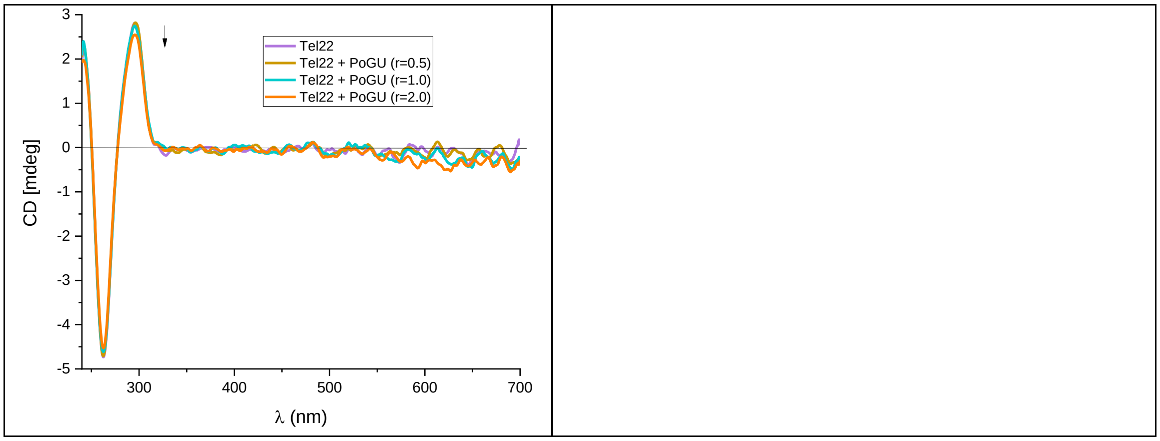

Figure S33. Changes in the CD spectrum of Tel22 (c =1 × 10^{-6} mol dm^{-3}) upon addition of **PoGU** (left) at different molar ratios r = [compound] / [polynucleotide], pH = 7.0, sodium cacodylate buffer, I =0.1 mol dm^{-3}

3. Biological Activity

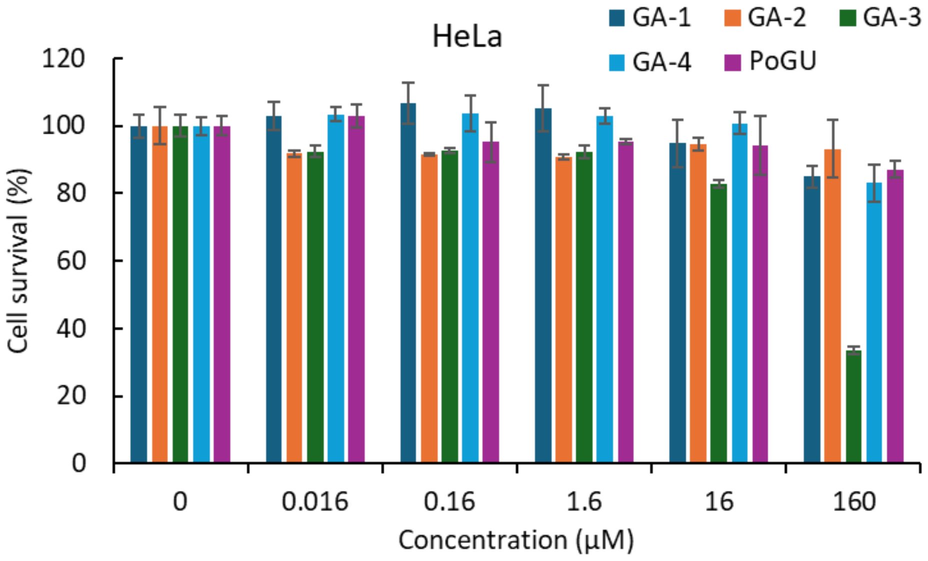

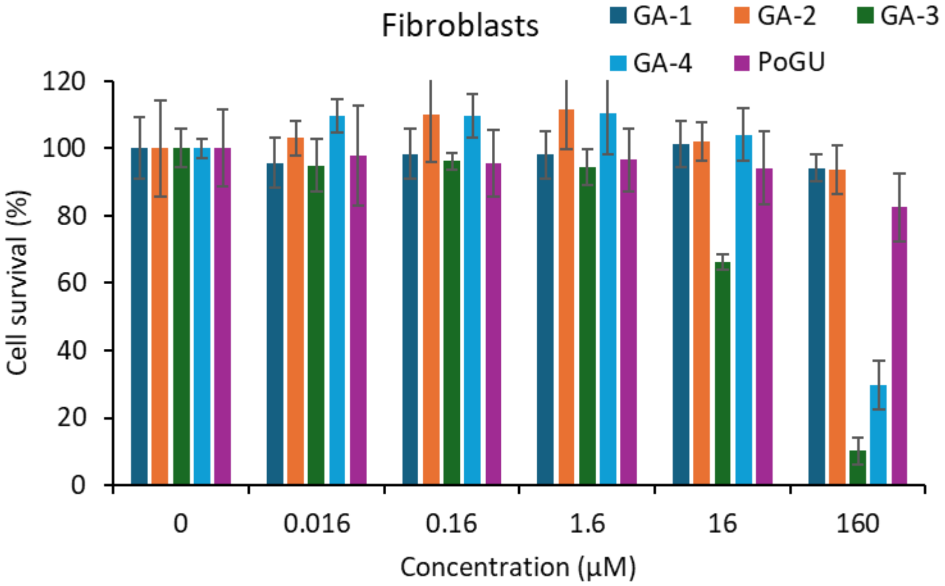

Figure S34. Dose-response profiles for compounds **GA1-GA4** and **PoGU** tested *in vitro* on HeLa cells (up) and Fibroblasts (down). Exponentially growing cells were treated during 72-hrs period. Metabolic activity was determined by MTT assay. Data represent mean values ± standard deviation (SD) of quadruplicate. The experiments were repeated at least three times

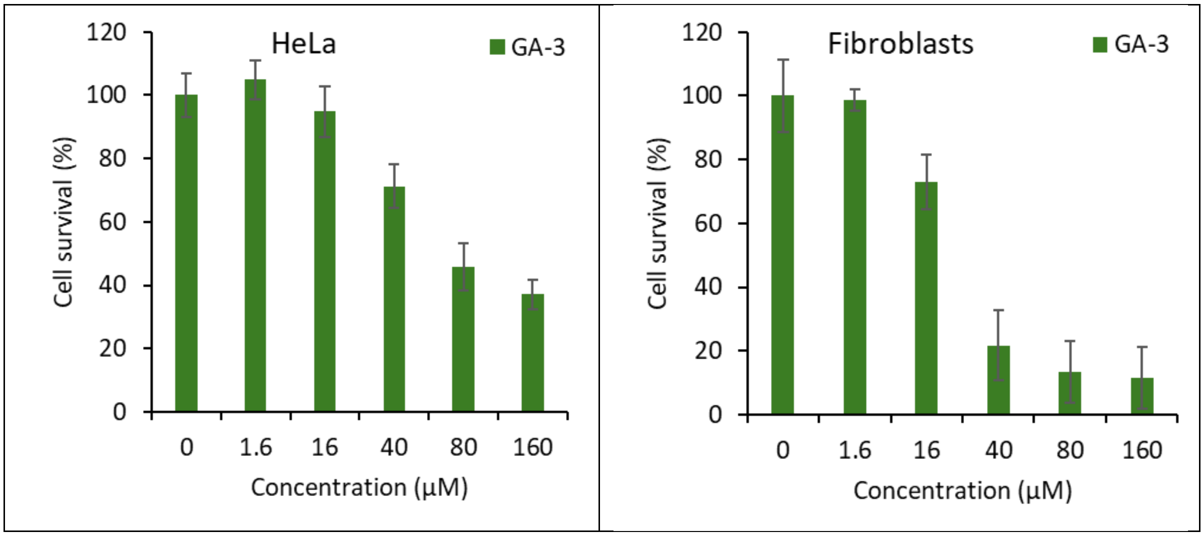

Figure S35. Dose-response profiles for compound **GA3** tested *in vitro* on HeLa cell line (left) and Fibroblasts (right). Exponentially growing cells were treated during 72-hrs period. Metabolic activity was determined by MTT assay. Data represent mean values ± standard deviation (SD) of quadruplicate. The experiments were repeated at least three times.

4. NMR and mass spectra of GA1-GA4

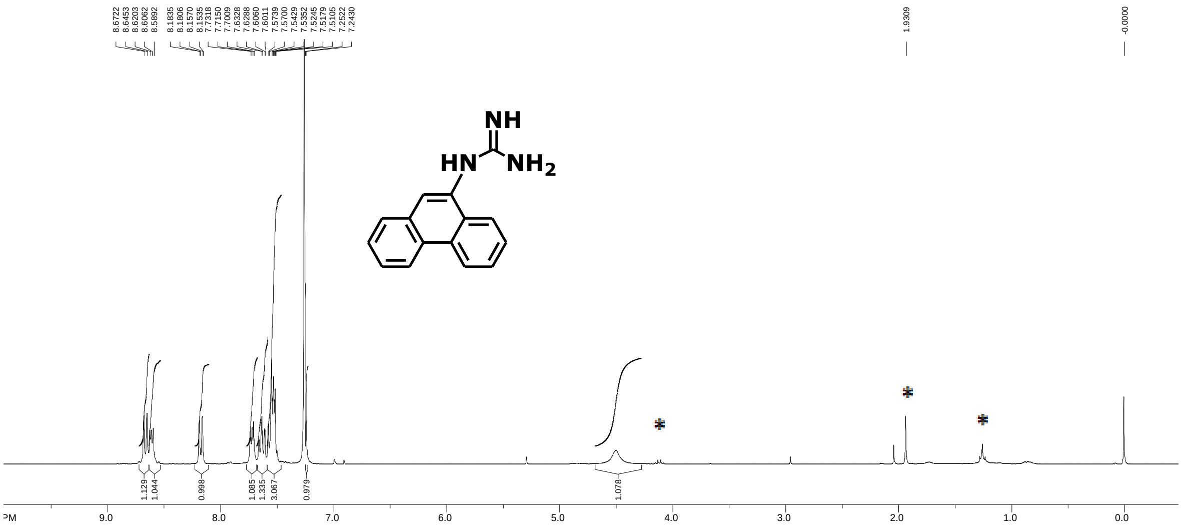

Figure S36. ^{1}H-NMR spectrum of **GA1** in $CDCl_3$ (*=Ethyl acetate)

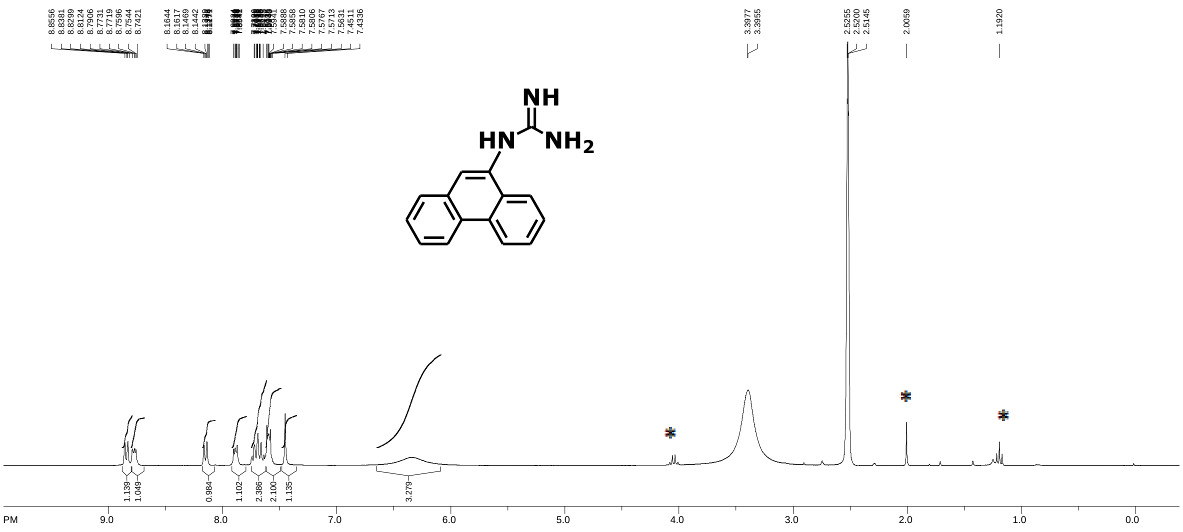

Figure S37. ^{1}H-NMR spectrum of **GA1** in DMSO-d_6

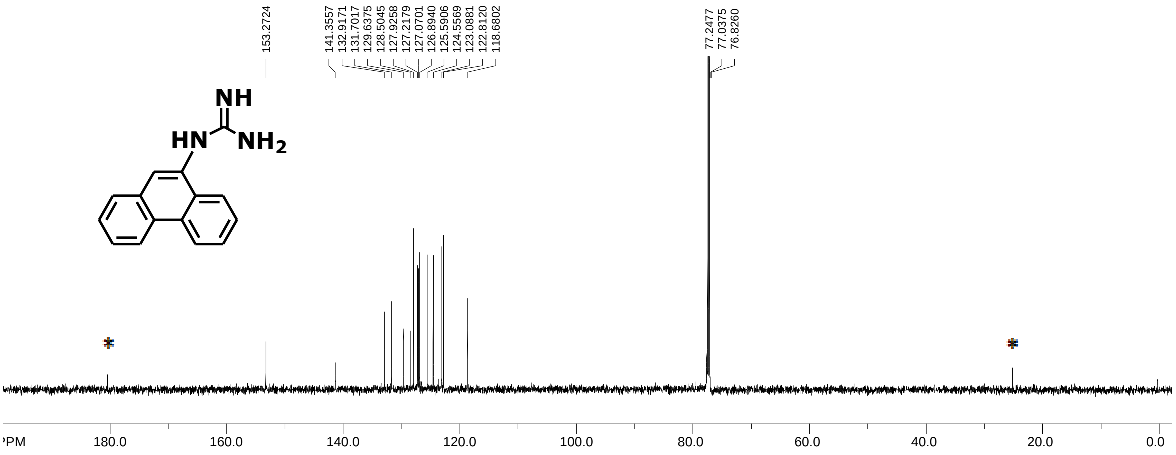

Figure S38. ^{13}C-NMR spectrum of **GA1** in $CDCl_3$

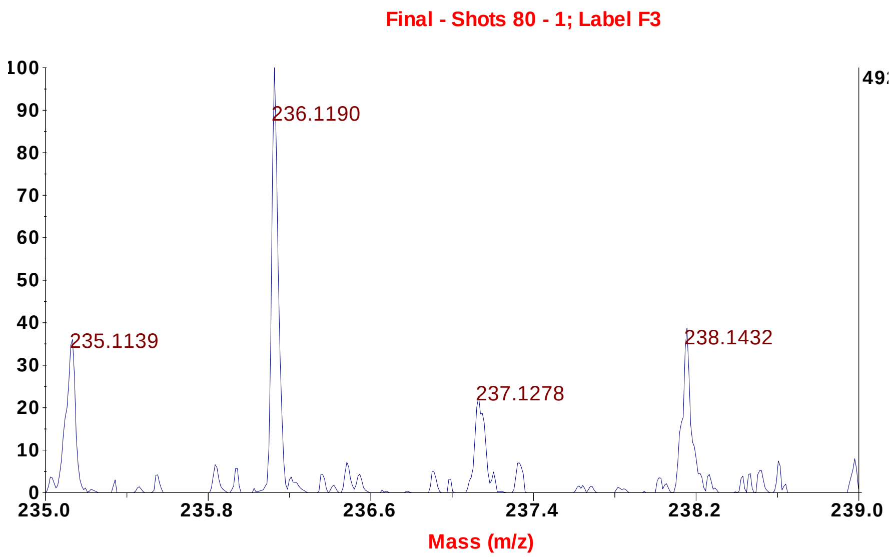

Figure S39. Mass spectrum of **GA1** in the range *m/z* 235-239

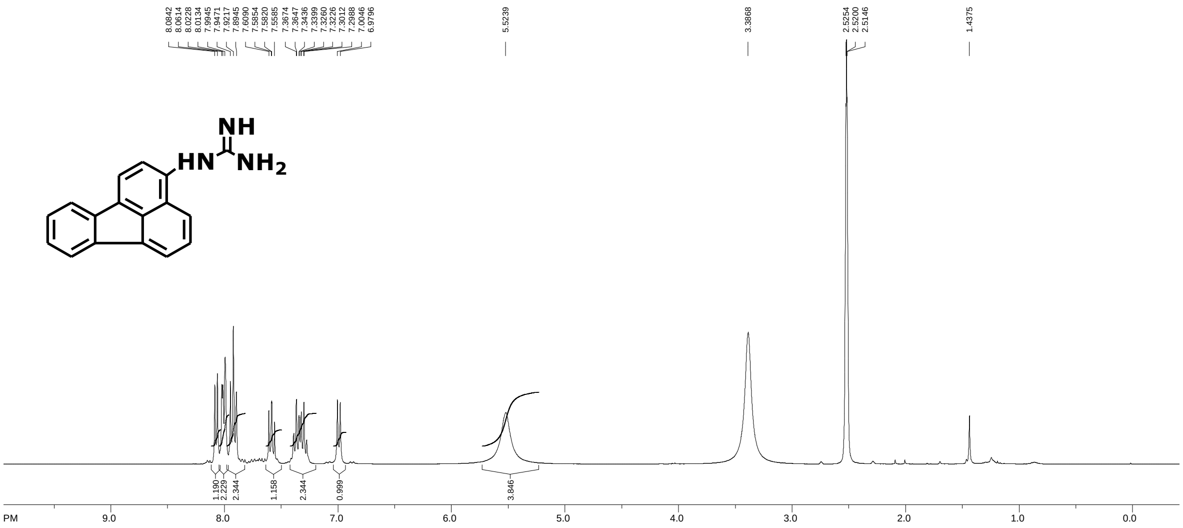

Figure S40. ^{1}H-NMR spectrum of **GA2** in DMSO-d_6

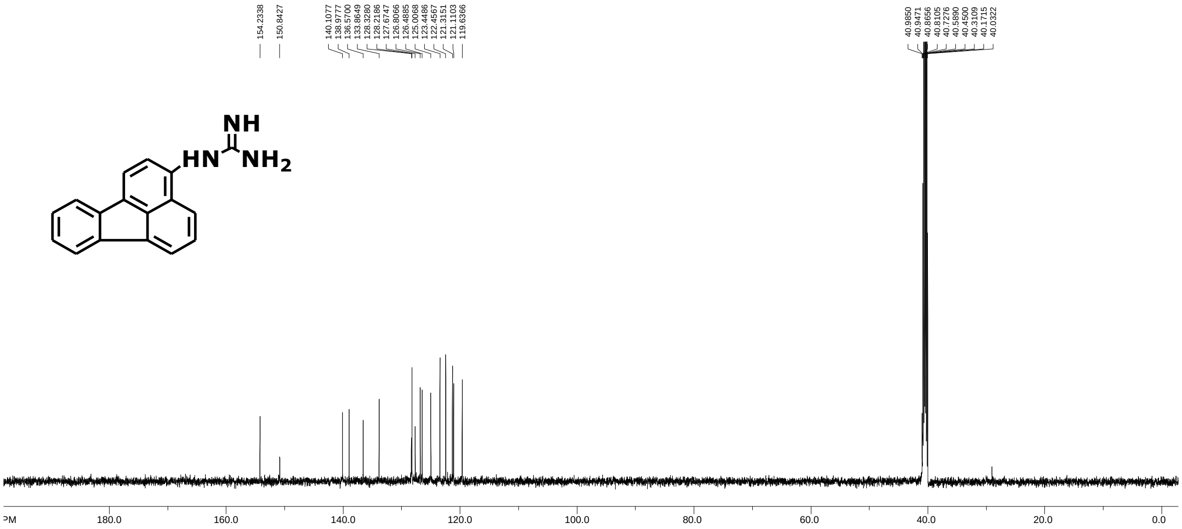

Figure S41. ^{13}C-NMR spectrum of **GA2** in DMSO-d_6

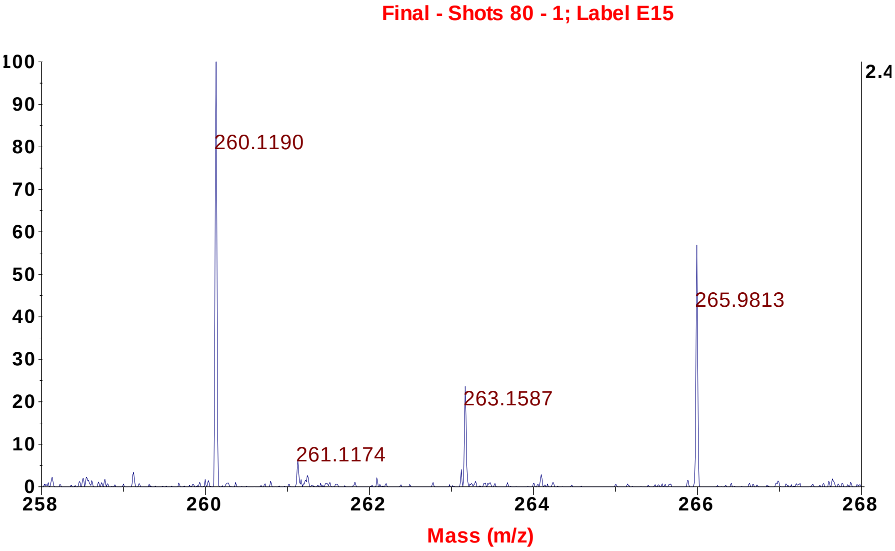

Figure S42. Mass spectrum of **GA2** in the range *m/z* 258-268

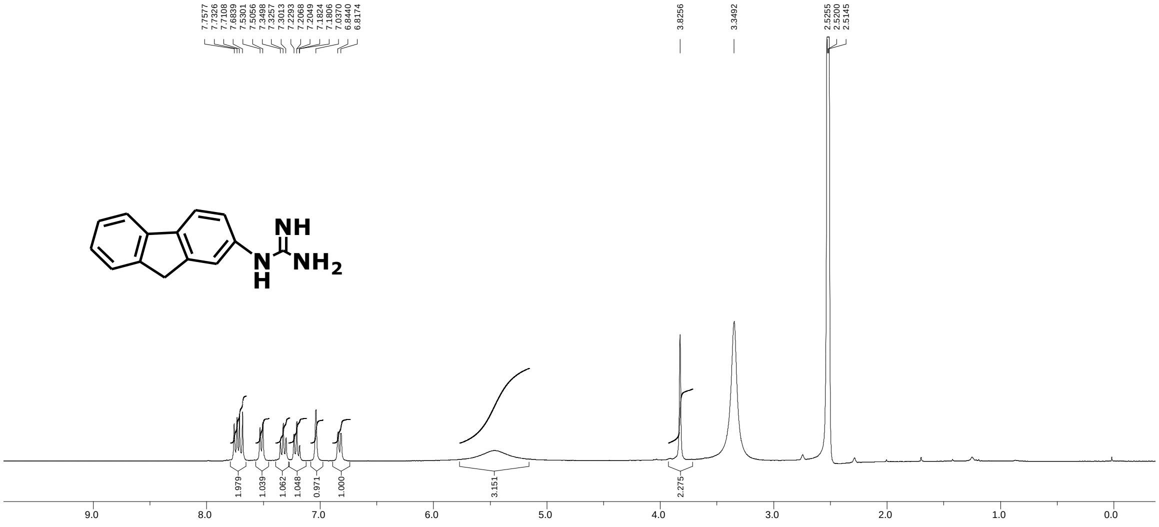

Figure S43. ^{1}H-NMR spectrum of **GA3** in DMSO-d_6

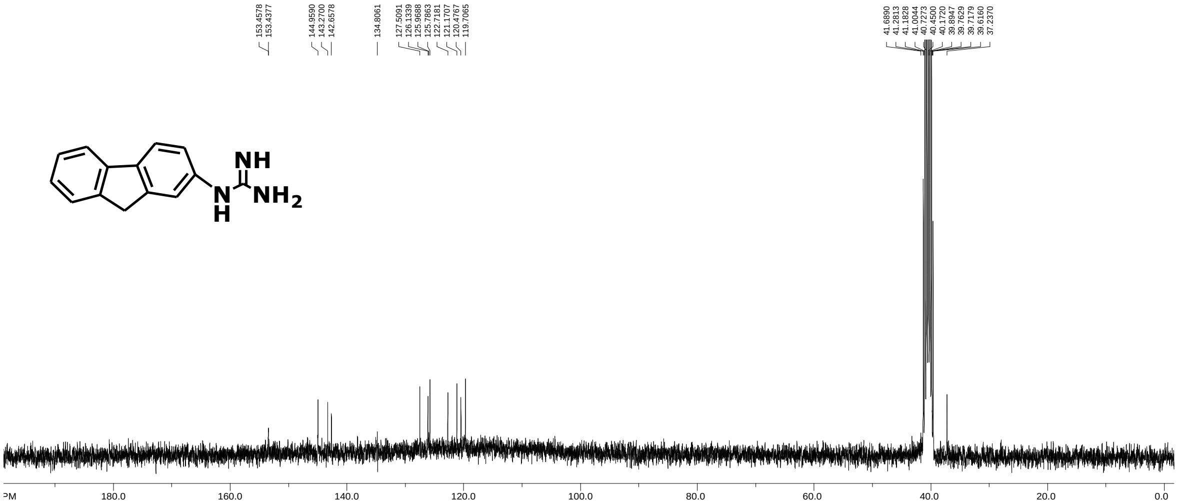

Figure S44. ^{13}C-NMR spectrum of **GA3** in DMSO-d_6

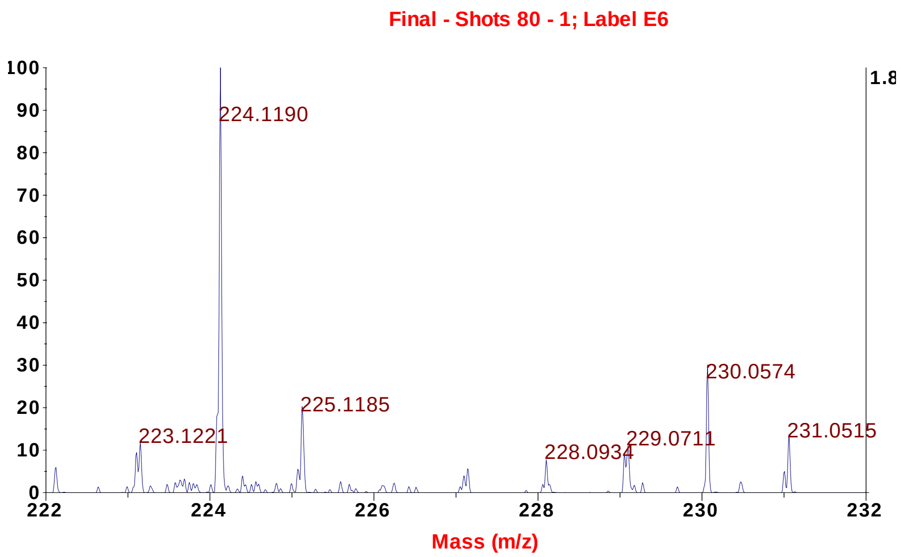

Figure S45. Mass spectrum of **GA3** in the range *m/z* 222-232

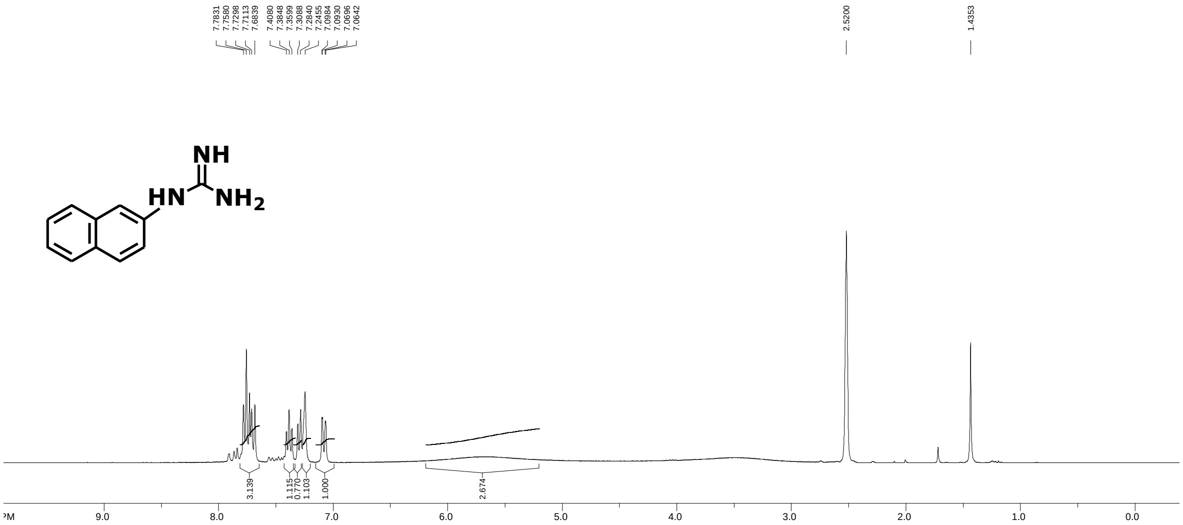

Figure S46. ^{1}H-NMR spectrum of **GA4** in DMSO-d_6

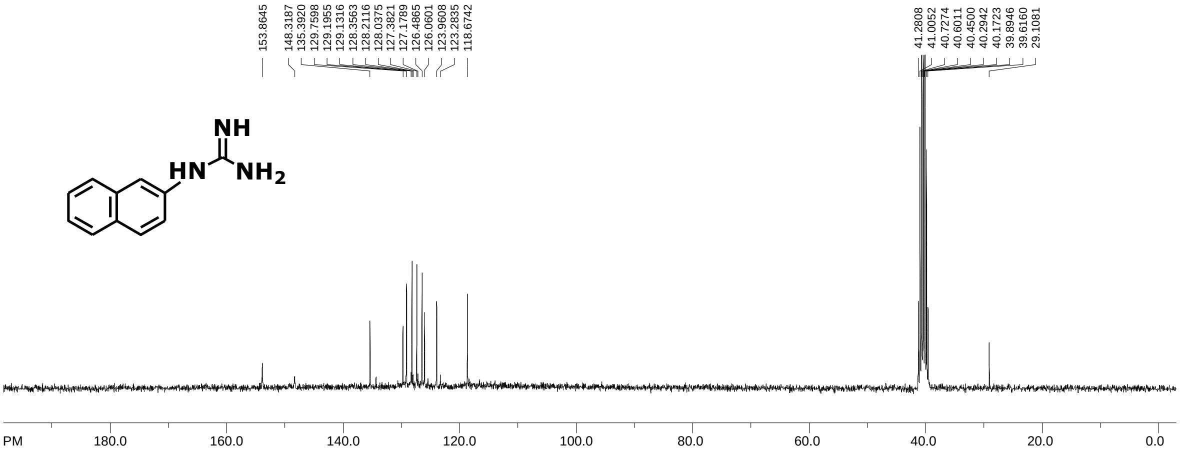

Figure S47. ^{13}C-NMR spectrum of **GA4** in DMSO-d_6

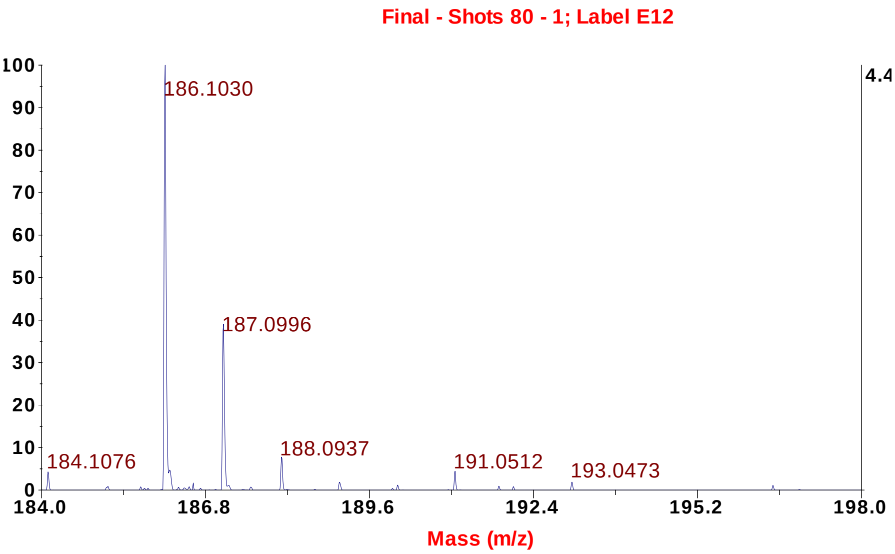

Figure S48. Mass spectrum of **GA4** in the range *m/z* 184-198

[1] M. Đud, Z. Glasovac, D. Margetić, I. Piantanida, *New J. Chem.* **2020**, *44*, 11537-11545.

[2] W. Saenger, *Principles of Nucleic Acid Structure*, Springer-Verlag: New York, **1983**, 226.

[3] Cantor, C.R. *Techniques for the Study of Biological Structure and Function*; Schimmel, P.R., Ed.; W.H. Freeman: San Francisco, CA, USA, 1980; pp. 399–404.

[4] J. D. McGhee, P. H. von Hippel, *J. Mol. Biol.,* **1974**, *86,* 469-489.

[5] G. Scatchard, *Ann. N. Y. Acad. Sci.* **1949**, *51,* 660-672.
